# Peripheral BDNF–TrkB Expression and Vitamin D Status in Depression, Prediabetes and Type 2 Diabetes: A Cross-Sectional Biomarker Study

**DOI:** 10.3390/biomedicines14092106

**Published:** 2026-09-18

**Authors:** Oleksandr Pavlovych, Iryna Kamyshna, Larysa Pavlovych, Oleh Lushchak, Iryna Halabitska, Pavlo Petakh, Oleksandr Kamyshnyi

**Affiliations:** 1 Department of Physiology Named After Ya.D. Kirshenblat, Bukovinian State Medical University, 58002 Chernivtsi, Ukraine; docmels@gmail.com; 2 Department of Medical Rehabilitation, I. Horbachevsky Ternopil National Medical University, 46001 Ternopil, Ukraine; kamyshna_ii@tdmu.edu.ua; 3 Department of Clinical Immunology, Allergology and Endocrinology, Bukovinian State Medical University, 58000 Chernivtsi, Ukraine; yapavlovych@gmail.com; 4 Kyiv School of Economics, 02000 Kyiv, Ukraine; oleh.lushchak@cnu.edu.ua (O.L.); pavlo.petakh@uzhnu.edu.ua (P.P.); 5 Department of Therapy and Family Medicine, I. Horbachevsky Ternopil National Medical University, 46001 Ternopil, Ukraine; halabitska@tdmu.edu.ua; 6 Department of Biochemistry and Pharmacology, Uzhhorod National University, 88000 Uzhhorod, Ukraine; 7 Department of Microbiology, Virology, and Immunology, I. Horbachevsky Ternopil National Medical University, 46001 Ternopil, Ukraine

**Keywords:** BDNF, NTRK2 (TrkB), NGF, 25-hydroxyvitamin D, prediabetes, type 2 diabetes, depression, anxiety, gene expression, metabolic–affective comorbidity, peripheral biomarkers

## Abstract

**Background/Objectives**: Depression and anxiety cluster with dysglycaemia along a prediabetes–type 2 diabetes continuum, but the signatures of affective and metabolic burden are unclear. We examined whether neurotrophin-related gene expression and 25(OH)D status track symptoms, parameters, or both. **Methods**: A cross-sectional study of 200 adults (*n* = 50 each: type 2 diabetes, prediabetes, depressive–anxiety, apparently healthy controls) was conducted. Anthropometry, glycaemic and lipid indices, PHQ-9, GAD-7, serum 25(OH)D and RT-qPCR expression of BDNF, NGF, NTRK1 and NTRK2 in whole peripheral blood were assessed. Multivariable regression, dominance analysis and pre-specified sensitivity analyses were applied. **Results**: BDNF, NGF and NTRK2 expression progressively decreased from controls to prediabetes and type 2 diabetes and was lowest in the depressive–anxiety group (*p* < 0.001); NTRK1 expression did not differ. Peripheral BDNF was more closely associated with PHQ-9 and 25(OH)D than with glycaemic or anthropometric indices. Psychiatric variables accounted for approximately 81% of the variance explained by the multivariable model (apparent R^2^ = 0.257), corresponding to approximately 21% of the total observed variance in log(BDNF). **Conclusions**: Peripheral BDNF and NTRK2 mRNA and 25(OH)D status were associated with depressive symptom burden more strongly than with metabolic parameters across a prediabetes–type 2 diabetes continuum. These associations describe an exploratory biomarker pattern; they do not establish altered signalling activity, causality or clinical utility, which require prospective validation.

## 1. Introduction

Depression and anxiety disorders are significantly more common in people with type 2 diabetes (T2DM) and its early dysmetabolic forms [1,2,3]. Such comorbidity worsens glycaemic control, adherence to treatment, and long-term prognosis [4,5,6,7]. The relationship between metabolic and affective disorders is bidirectional and is only partially explained by the psychological burden associated with chronic disease [8,9,10,11]. This suggests the existence of shared biological pathways that may begin to operate even before the development of clinically significant pathology [4,12,13,14]. The identification of such mechanisms is an important step towards the transition from a reactive approach in psychiatric care to a biologically based risk stratification in metabolic medicine.

One of the most promising candidates for the role of such mechanisms is neurotrophins [14,15,16,17]. Brain-derived neurotrophic factor (BDNF) and nerve growth factor (NGF) regulate neuronal survival, synaptic plasticity [18,19,20], and, as recent studies have shown, are involved in the regulation of systemic energy metabolism [21,22,23]. They exert their effects through the tyrosine kinase receptors TrkB (NTRK2) and TrkA (NTRK1), respectively [24,25,26]. According to the neurotrophic hypothesis of affective disorders, a decrease in the activity of the BDNF–TrkB signalling pathway contributes to the development of synaptic atrophy characteristic of depression [27,28,29,30]. In addition, peripheral levels of BDNF are one of the most reproducible laboratory markers of depressive states [31,32,33]. At the same time, BDNF affects insulin sensitivity and appetite regulation [34,35,36,37], which makes it a potential molecular link between affective and metabolic disorders.

Vitamin D plays an important role in this signalling system. Its receptors are widely distributed in brain tissues [38,39,40,41], and the active form of vitamin D, 1,25-dihydroxyvitamin D, stimulates transcription of the *BDNF* gene [42,43,44]. Vitamin D deficiency is frequently observed in metabolic diseases and is independently associated with the development of depression [45,46,47]. However, although reduced peripheral BDNF expression and low vitamin D status have each been associated with depression and metabolic disease, substantially less is known about their relative relationships when affective symptom burden, dysglycaemia, adiposity and neurotrophin-related gene expression are assessed simultaneously within the same analytical framework. In particular, it remains unclear whether peripheral BDNF- and NTRK2-related transcriptional changes are more closely associated with the severity of affective symptoms or with metabolic abnormalities, and whether these associations remain after accounting for the overlap between psychiatric and metabolic characteristics.

A further reason to examine these associations across the prediabetes–type 2 diabetes continuum, rather than in overt T2DM alone, is that dysglycaemia is a graded exposure rather than a binary state. Impaired glucose tolerance is already accompanied by low-grade inflammation, insulin resistance and endothelial dysfunction, and it carries an elevated risk of depression that precedes the diagnosis of T2DM. Including prediabetes as a distinct group therefore allows any BDNF-, NTRK2- or vitamin D-related signal that emerges early—before the metabolic phenotype is fully expressed—to be distinguished from a signal confined to established diabetes.

The present study therefore extends previous work by integrating clinical phenotyping, psychometric assessment, metabolic characterisation, serum 25(OH)D measurement and peripheral expression analysis of *BDNF*, *NGF*, *NTRK1* and *NTRK2* in four clinically distinct groups: healthy controls, individuals with prediabetes, patients with type 2 diabetes, and individuals with depressive–anxiety disorders. Rather than assuming that metabolic and psychiatric dimensions are fully separable at the group level, we examined their relative statistical contributions and overlap at the participant level. Specifically, we investigated whether: (i) peripheral expression of neurotrophin-related genes differs across the metabolic–affective spectrum; (ii) these expression measures are more strongly associated with affective symptom burden, metabolic characteristics or vitamin D status; (iii) psychiatric and metabolic variables provide differential statistical contributions to variation in peripheral BDNF mRNA expression; and (iv) the observed associations remain robust after multivariable and sensitivity analyses.

The present study was conducted in a population living under conditions of ongoing war, representing a specific and insufficiently studied psychosocial and environmental context. Although associations of BDNF with major depressive disorder and type 2 diabetes, as well as associations of low vitamin D status with depressive disorders and type 2 diabetes, have been previously reported [48,49,50,51], the expression of *BDNF*, *NGF*, *NTRK1* and *NTRK2* together with serum 25(OH)D status and metabolic and psychoemotional characteristics has not, to the best of our knowledge, been previously investigated in a population living under wartime conditions. Thus, the novelty of the present study lies primarily in the characterisation of these neurotrophin-related and metabolic–affective relationships within this specific and previously insufficiently studied population. Importantly, we do not claim that the individual associations between BDNF, vitamin D, depression and metabolic abnormalities are themselves novel. Rather, our study provides a comparative assessment of these relationships in the context of prolonged war-related psychosocial and environmental stress, while also examining the relative contribution of affective and metabolic dimensions to peripheral BDNF expression.

## 2. Materials and Methods

### 2.1. Study Design and Participants

**Figure 1:**
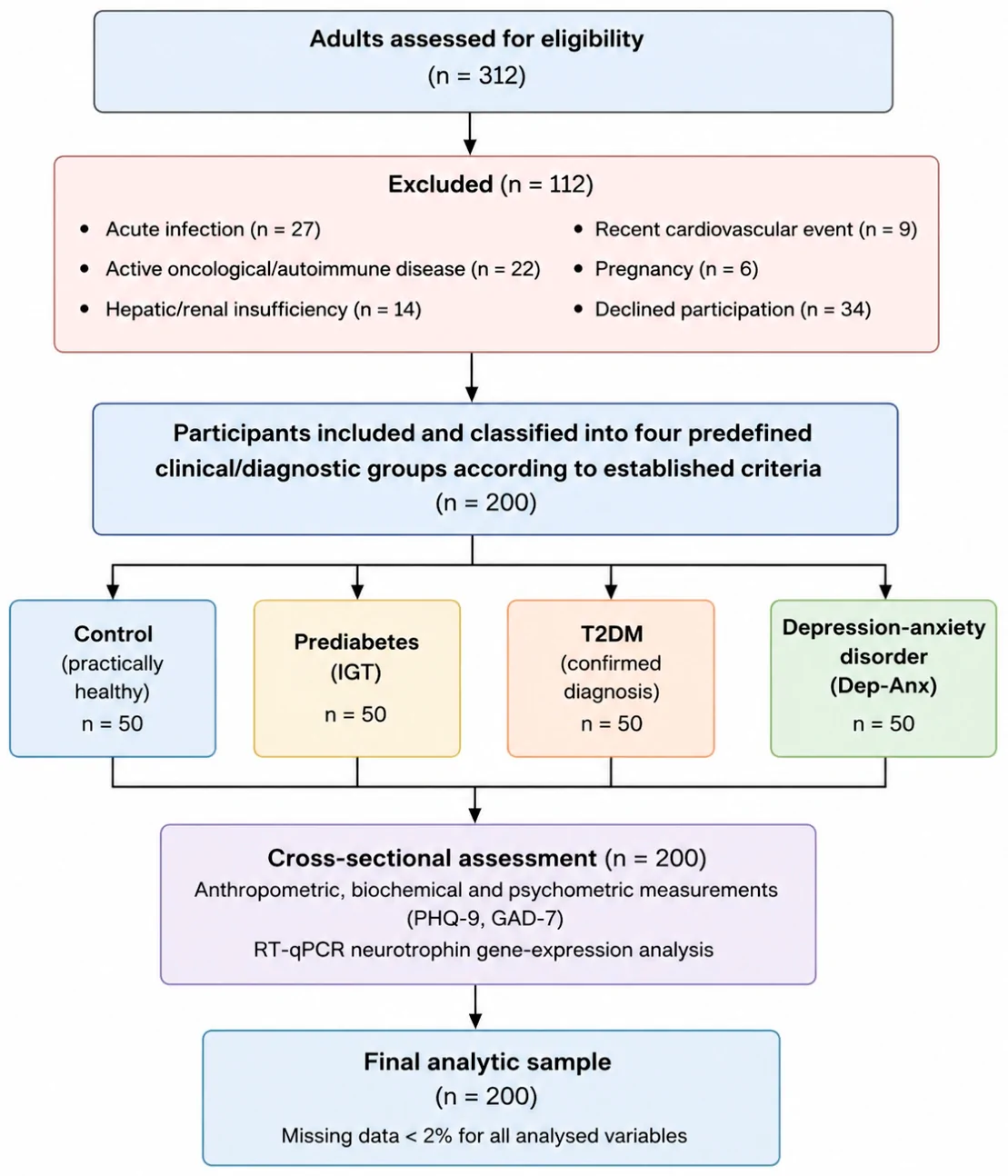
Participant flow and allocation. Of 312 individuals assessed for eligibility, 112 were excluded (acute infection *n* = 27; active oncological/autoimmune disease *n* = 22; hepatic/renal insufficiency *n* = 14; recent cardiovascular event *n* = 9; pregnancy *n* = 6; declined participation *n* = 34). In total, 200 participants were included and allocated consecutively within each of four predefined groups (*n* = 50 each) until the planned sample size was reached: control, prediabetes, type 2 diabetes, and depressive–anxiety. Missing data were <2% for all analysed variables. Because metabolic and affective dimensions overlap across groups, their relative associations with peripheral gene expression were examined using multivariable and variance-partitioning analyses.

The comparative cross-sectional study included 200 adult patients and apparently healthy controls recruited from the outpatient endocrinology department of the Chernivtsi Regional Clinical Hospital from September 2024 to December 2025 (Figure 1). According to the study protocol, the participants were divided into four groups of 50 individuals each: (1) patients with type 2 diabetes mellitus diagnosed according to the criteria of the American Diabetes Association [52]; (2) patients with prediabetes (impaired glucose tolerance); (3) patients with depressive–anxiety disorders in the absence of clinical and laboratory signs of carbohydrate metabolism disorders; (4) apparently healthy controls without established somatic and mental pathology, who formed the control group. The four-group design was intended to provide clinically contrasting patterns of metabolic status and affective symptom burden. Because metabolic and psychiatric characteristics may coexist within the same participants, the four clinical groups were not treated as mutually exclusive representations of “metabolic-only” or “psychiatric-only” states. The group structure was used to provide clinically contrasting phenotypes, whereas participant-level symptom scores and metabolic measurements were used to characterise the continuous dimensions of affective and metabolic burden. Multivariable regression and variance-partitioning analyses were therefore applied to quantify the relative statistical associations of these measured dimensions with peripheral gene expression. These analyses were not intended to eliminate the underlying overlap between phenotypes or to establish independent causal effects.

The study included individuals aged 18 years and older who provided written informed consent to participate and met the criteria for one of the four study groups. The type 2 diabetes group included patients with diagnoses established according to the American Diabetes Association (ADA) criteria. The prediabetes group included patients with impaired glucose tolerance. The depressive–anxiety disorder group consisted of participants with clinically significant symptoms of depression and/or anxiety in the absence of clinical and laboratory signs of impaired carbohydrate metabolism. Diagnoses in this group were established by a board-certified psychiatrist according to ICD-10 criteria (F32 depressive episode, F33 recurrent depressive disorder, and/or F41 anxiety disorders); PHQ-9 and GAD-7 were used to quantify symptom severity rather than as standalone diagnostic instruments. Participants with a lifetime diagnosis of bipolar disorder, schizophrenia-spectrum disorders or substance-use disorders were excluded. Illness duration, ICD-10 diagnostic distribution and treatment status of this group are summarised in Supplementary Table S2b. The control group included apparently healthy controls without established metabolic or psychiatric diseases.

Exclusion criteria were pregnancy or breastfeeding, acute infectious or inflammatory diseases, active oncological or autoimmune diseases, severe hepatic or renal failure, acute cardiovascular events within the last six months, and any conditions that could affect participation in the study or interpretation of the results.

Information on current medication use was collected from medical records and a structured questionnaire for all study participants. Use of antidepressants, anxiolytics, antipsychotics, hypoglycaemic therapy (metformin, insulin, glucagon-like peptide-1 (GLP-1) receptor agonists), lipid-lowering therapy (statins), and vitamin D supplementation were recorded. This information was collected to characterise the study cohort and to account for the possible impact of medication use when interpreting the results, but these variables were not included as covariates in the main statistical models.

The study protocol was approved by the Bioethics Commission of the Bukovinian State Medical University of the Ministry of Health of Ukraine (protocol No. 3 dated 17 October 2024). The study was conducted in accordance with the ethical principles of the Declaration of Helsinki of the World Medical Association and its subsequent revisions. Before inclusion in the study, all participants received comprehensive information about its purpose and design and provided written informed consent to participate.

### 2.2. Clinical, Biochemical and Psychometric Assessment

All participants had their anthropometric measurements taken, including body weight and body mass index (BMI). Carbohydrate metabolism was assessed by glycated haemoglobin (HbA1c), fasting plasma glucose, 2 h oral glucose tolerance test, and HOMA-IR insulin resistance index. Lipid profile included total cholesterol, triglycerides, low-density lipoprotein cholesterol (LDL-C), and high-density lipoprotein cholesterol (HDL-C). Biochemical parameters were determined on a Cobas c311 automated biochemical analyser (Roche Diagnostics, Mannheim, Germany) using the manufacturer’s original reagents according to the manufacturer’s instructions and standard internal quality control procedures. LDL-C was calculated using the Friedewald equation when applicable, based on total cholesterol, HDL-C and triglyceride concentrations.

Serum 25-hydroxyvitamin D (25(OH)D) concentration was determined by electrochemiluminescent immunoassay on a Cobas e411 automated analyser (Roche Diagnostics, Mannheim, Germany) using Elecsys Vitamin D total II test systems. Vitamin D status was assessed according to generally accepted threshold values: adequate level—30–50 ng/mL, insufficiency—20–29 ng/mL, deficiency—less than 20 ng/mL [53,54].

The psychoemotional state of the participants was assessed using validated international psychometric instruments. The severity of depressive symptoms was determined using the Patient Health Questionnaire-9 (PHQ-9) [55,56], anxiety by the Generalized Anxiety Disorder-7 (GAD-7) [57,58], subjective psychological well-being by the World Health Organization Well-Being Index (WHO-5) [59,60], and diabetes-related distress by the Problem Areas in Diabetes (PAID) scale [61,62]. The risk of developing type 2 diabetes was assessed using the Finnish Diabetes Risk Score (FINDRISC) [63,64]. For the interpretation of the results of all psychometric scales, generally accepted severity categories were used according to the recommendations of their developers.

### 2.3. Neurotrophin Expression

Peripheral expression of BDNF, NGF, NTRK1, and NTRK2 genes was determined by real-time reverse transcription quantitative polymerase chain reaction (RT-qPCR). Whole peripheral blood samples obtained from all study participants were used for analysis. Because RNA was extracted from whole peripheral blood, the measured transcript levels represent composite signals derived from circulating blood-cell populations. Differential leukocyte or other cellular composition was not quantified and therefore could not be included as a covariate. Accordingly, between-group differences in peripheral gene expression may partly reflect differences in blood-cell composition and should be interpreted as whole-blood transcriptional associations rather than cell-specific expression changes. Total RNA was isolated using TRIzol™ Reagent (Cat. No. 15596026, Thermo Fisher Scientific, Waltham, MA, USA) according to the manufacturer’s instructions. The concentration and purity of the isolated RNA were assessed spectrophotometrically, after which equal amounts of RNA were reverse transcribed using the RevertAid™ First Strand cDNA Synthesis Kit (Cat. No. K1622, Thermo Fisher Scientific, Waltham, MA, USA) to produce complementary DNA (cDNA).

The mRNA expression levels of BDNF, NGF, NTRK1, and NTRK2 genes were determined by RT-qPCR on a CFX96 Real-Time PCR Detection System (Bio-Rad Laboratories, Hercules, CA, USA; cat. no. 185-5096) using the Maxima SYBR Green/ROX qPCR Master Mix (2×, Thermo Scientific, Waltham, MA, USA; cat. no. K0221). For each gene under study and the reference gene ACTB, individually synthesised forward and reverse primers were used (synthesis scale—25 nmol, purification by desalting method). All reactions were performed in three technical replicates. Primer sequences, amplicon lengths, amplification efficiencies and R^2^ values for all target and reference genes are provided in Supplementary Table S1.

Primer specificity was confirmed by BLAST analysis and by single-peak melting curves. Amplification efficiencies calculated from 5-point serial dilution standard curves were 92–104% (R^2^ ≥ 0.99) for all assays (Supplementary Table S1).

Total RNA concentration and purity were assessed spectrophotometrically (NanoDrop; acceptance A260/A280 = 1.9–2.1, A260/A230 ≥ 2.0). RNA integrity was assessed by microcapillary electrophoresis (Agilent Bioanalyzer, Agilent Technologies, Santa Clara, CA, USA); RNA integrity number (RIN) was ≥ 7.5 for all samples. For reverse transcription, 1 μg of total RNA was used in a 20 μL reaction; 2 μL of cDNA was used per 20 μL qPCR reaction. Ct values for target and reference transcripts ranged from 22 to 34; no samples yielded undetermined Ct values.

Each run included no-template controls (NTC) and no-reverse-transcription (NRT) controls; an inter-run calibrator sample was included on every plate. Samples from all four groups were randomly distributed across PCR plates using stratified randomisation to avoid batch–group confounding. Laboratory personnel performing RNA extraction and RT-qPCR were blinded to participant group allocation; samples were labelled with numeric codes only. The stability of ACTB expression across the four groups was verified prior to normalisation (Kruskal–Wallis on raw Ct, *p* = 0.44; coefficient of variation of Ct < 3%). Reporting of the RT-qPCR methodology follows the MIQE guidelines (File S1).

Amplification was performed according to the following protocol: initial denaturation at 95 °C for 10 min, followed by 45 cycles, which included denaturation at 95 °C for 15 s, annealing of primers at 60 °C for 40 s, and elongation at 72 °C for 40 s. The specificity of amplification was confirmed by melting curve analysis. The relative level of gene expression was calculated by the 2^−ΔΔCt^ method with normalisation to the expression of the reference gene ACTB.

### 2.4. Statistical Analysis

The primary outcome was the association between peripheral BDNF mRNA expression and depression severity (PHQ-9), adjusted for 25(OH)D, age and sex. Secondary outcomes were the analogous associations for NGF and NTRK2, and the relative contributions of psychiatric and metabolic variables to variance in log(BDNF). Confirmatory analyses comprised the between-group comparisons of gene expression (Kruskal–Wallis with Dunn post hoc) and the multivariable OLS regression of log(BDNF). Robust regression, quantile regression, equivalence testing, mediation decomposition, dominance analysis and network analysis were prespecified as exploratory. Benjamini–Hochberg FDR correction was applied within two families: (i) between-group comparisons; (ii) the Spearman correlation matrix (136 pairs). The equivalence margin of ±0.15 for standardised regression coefficients was chosen a priori as a conventional threshold for a negligible practical effect. Sample size was determined a priori using Cohen’s tables for partial correlation: with α = 0.05 (two-sided) and power = 0.90, detecting a partial correlation of ρ = 0.25 requires *n* = 168; the achieved sample of *n* = 200 exceeds this requirement and provides > 95% power for the primary hypothesis. Medication use and vitamin D supplementation were deliberately not included as covariates in the primary multivariable model because they are downstream of, and partly determined by, the exposures of interest (psychiatric diagnosis, glycaemic status and vitamin D status); adjusting for them in a primary analysis would produce collider-like bias. Instead, they were pre-specified as covariates in sensitivity analyses (Supplementary Table S3), where the primary associations were preserved. Reporting follows the STROBE Statement for cross-sectional studies (File S2); complete analytical code is provided as File S3.

Statistical analysis of the data was performed using the Python 3.12 software environment (SciPy, statsmodels, scikit-learn, pingouin, scikit-posthocs, networkx). Since most of the indicators did not correspond to a normal distribution (according to the Shapiro–Wilk test, *p* < 0.05 in most groups), the Kruskal–Wallis test was used to compare groups with the calculation of the effect size ε^2^ and subsequent pairwise comparisons using the Dunn test. The χ^2^ test was used to analyse the relationships between categorical variables with the calculation of the Cramer’s V coefficient. Correction for multiple comparisons was performed using the Benjamini–Hochberg method with false discovery rate (FDR) control. Spearman correlation analysis was used to assess the relationships between quantitative indicators, and, taking into account the influence of potential confounding factors, Spearman partial correlation analysis.

Depression severity (PHQ-9) was modelled by ordinary least squares (OLS) with heteroscedasticity-consistent (HC3) standard errors and z-standardised predictors. To examine the statistical correlates of variation in BDNF variance directly, log(BDNF) was modelled against a psychiatric block (PHQ-9, GAD-7) and a metabolic block (BMI, HbA1c, HOMA-IR) with age as a covariate; hierarchical entry in both orders yielded incremental R^2^ (ΔR^2^) attributable to each block, and general dominance analysis averaged each predictor’s incremental R^2^ across all 2^6^ possible orderings (a Shapley-value decomposition of R^2^) [65,66]. For internal validation of the models, a bootstrap procedure (1000 replicates) was used using the Harrell optimism method, which allowed us to obtain an adjusted value of the coefficient of determination (R^2^). To assess the robustness of the results to violations of the assumptions regarding the data distribution, Huber M-estimation and median (quantile, τ = 0.5) regression were additionally used. For metabolic predictors, a two-tailed equivalence test (TOST) was performed with a limit of ±0.15 for standardised regression coefficients. The moderating effect of body mass index was assessed by including the interaction term PHQ-9 × BMI in the model and analysing simple slopes at BMI values ±1 standard deviation from the mean. To explore whether variation in log(BDNF) accounted for any part of the association between BMI or HbA1c and PHQ-9, we performed an exploratory product-of-coefficients decomposition with bootstrap estimation (5000 replicates) and computed 95% confidence intervals for the indirect effect. Given the cross-sectional design, this decomposition is interpreted as a partition of statistical associations rather than as a causal mediation analysis. Variance inflation factor (VIF) was determined to assess multicollinearity; in all models, VIF values were less than 5. Throughout the manuscript, R^2^ denotes the coefficient of determination, i.e., the proportion of variance in the outcome accounted for by the specified model. Adjusted R^2^ refers to the coefficient of determination corrected for model complexity. Bootstrap optimism-corrected R^2^ refers to the apparent R^2^ adjusted for optimism estimated using the Harrell bootstrap procedure.

## 3. Results

### 3.1. Cohort Characteristics and Metabolic–Psychometric Architecture

**Figure 2:**
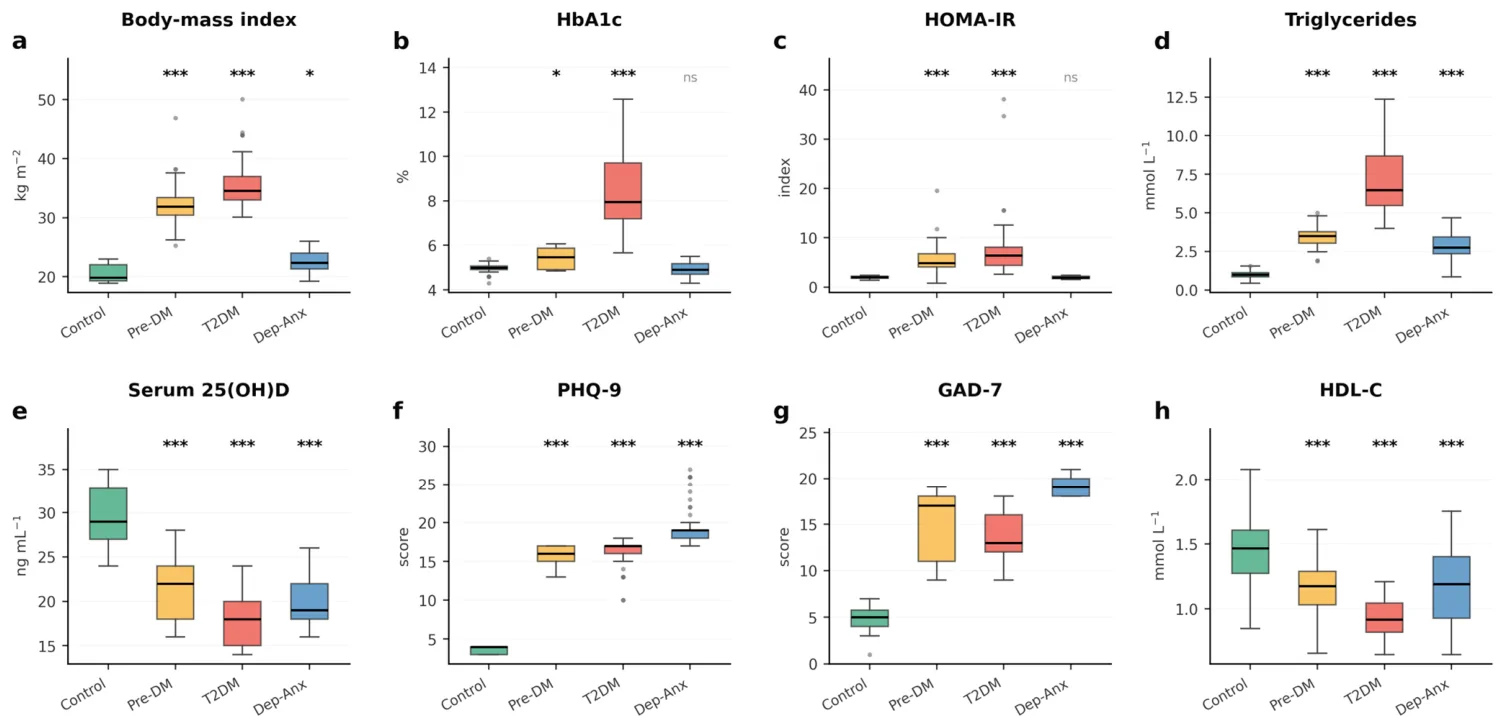
Metabolic and psychometric architecture of the four phenotypes. Box-and-jitter plots for (**a**) body-mass index, (**b**) HbA1c, (**c**) HOMA-IR, (**d**) triglycerides, (**e**) serum 25(OH)D, (**f**) PHQ-9, (**g**) GAD-7 and (**h**) HDL-cholesterol across control, prediabetes, T2DM and depressive–anxiety (Dep-Anx) groups (*n* = 50 each). Boxes show median and interquartile range; whiskers 1.5 × IQR; points are individual participants. All variables shown differ across groups at false-discovery-rate-corrected *p* < 0.001 (Kruskal–Wallis). The distributions also demonstrate substantial overlap between metabolic and affective dimensions, with elevated PHQ-9 and/or GAD-7 scores present in the prediabetes and T2DM groups. * indicates *p* < 0.05; *** indicates *p* < 0.001; ns indicates no statistically significant difference (*p* ≥ 0.05).

The four study groups (50 participants each) were comparable in age (median 38–42 years; Kruskal–Wallis test, *p* = 0.13), while statistically significant differences were found between them in all metabolic and psychometric parameters (Table 1; Figure 2). Extended demographic, lifestyle, medication, seasonal and psychometric characteristics of the cohort are provided in Supplementary Table S2. As predicted by the study design, the group of patients with type 2 diabetes mellitus recorded the highest values of obesity, carbohydrate metabolism disorders and atherogenic dyslipidaemia; in particular, the median HbA1c was 8.0%, HOMA-IR—6.4, and triglyceride level—6.5 mmol/L. Patients with prediabetes occupied an intermediate position between the control group and the type 2 diabetes mellitus group in most metabolic parameters.

In contrast, the group of patients with depressive–anxiety disorders was characterised by almost normal metabolic parameters (median BMI—22.4 kg/m^2^, HbA1c—4.9%, HOMA-IR—1.9), but had the highest severity of psychoemotional symptoms. The median values on the PHQ-9 and GAD-7 scales were 19 and 19 points, respectively, which indicated severe manifestations of depression and anxiety.

The concentration of 25(OH)D in serum gradually decreased from the control group (29 ng/mL) to the groups with metabolic and affective disorders (18–19 ng/mL). All pairwise comparisons between the control group and other groups according to Dunn’s criterion were statistically significant (*p* < 0.001), and vitamin D insufficiency or deficiency was detected in almost all participants outside the control group.

Large effect sizes were observed for key metabolic and psychometric indicators (ε^2^ = 0.59–0.86), confirming the high severity of intergroup differences. Importantly, the group with depressive–anxiety disorders had the greatest psychiatric symptoms, while remaining statistically similar to the control group in terms of BMI, HbA1c, and HOMA-IR. At the same time, the prediabetes group was characterised by significant metabolic disorders with significantly lower severity of psychoemotional disorders compared to the depression and anxiety group. Importantly, affective symptom burden was not confined to the depressive–anxiety group. Although the highest PHQ-9 and GAD-7 scores were observed in the depressive–anxiety group, participants with prediabetes and type 2 diabetes also showed substantial depressive and/or anxiety symptoms. Thus, the clinical groups represented distinct but overlapping metabolic and affective phenotypes rather than mutually exclusive metabolic-only and psychiatric-only categories.

This overlap was taken into account in subsequent analyses. Associations between peripheral neurotrophin-related gene expression and psychiatric or metabolic characteristics were therefore examined at the participant level using correlation, multivariable regression and variance-partitioning approaches rather than being inferred solely from between-group contrasts.

### 3.2. Neurotrophin Down-Regulation Is Graded and Maximal in the Affective Phenotype

**Figure 3:**
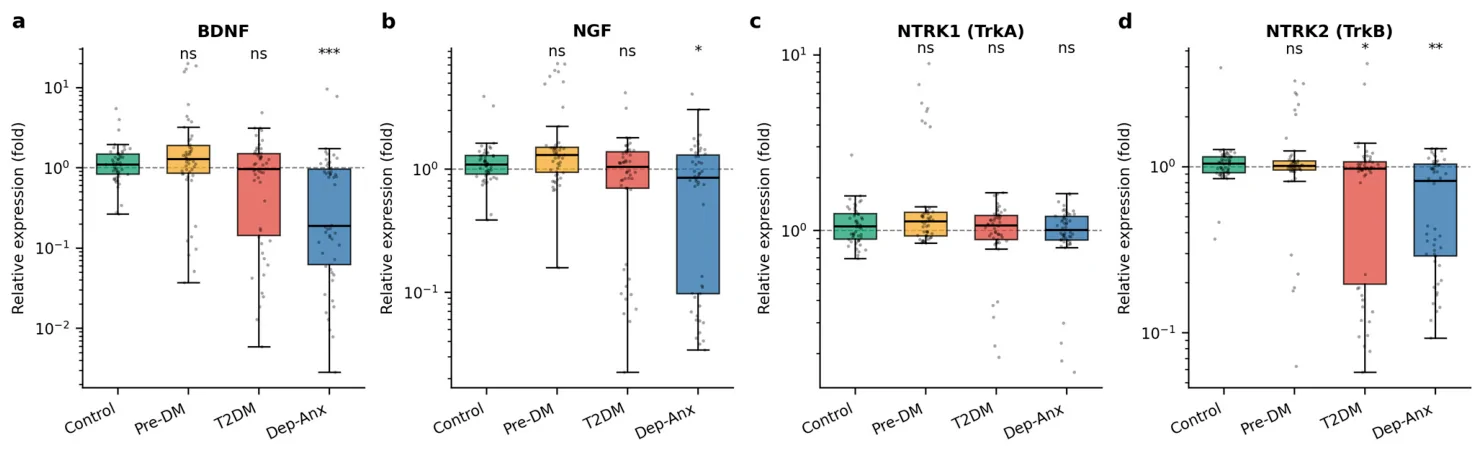
Coordinated changes in peripheral BDNF- and NTRK2-related gene expression (fold-change versus healthy reference, log scale) of (**a**) BDNF, (**b**) NGF, (**c**) NTRK1/TrkA and (**d**) NTRK2/TrkB. Dashed line marks unchanged expression (fold = 1). Asterisks denote Dunn post-hoc significance versus control after Benjamini–Hochberg correction (* indicates *p* < 0.05, ** indicates *p* < 0.01, *** indicates *p* < 0.001; ns, not significant). BDNF, NGF and NTRK2 transcripts were reduced, particularly in the depressive–anxiety group, whereas NTRK1 expression was relatively preserved.

Relative peripheral expression of BDNF, NGF and NTRK2 genes differed statistically significantly between the study groups (Kruskal–Wallis test: *p* = 1.9 × 10^−6^, *p* = 4.2 × 10^−4^ and *p* = 6.8 × 10^−4^, respectively; ε^2^ = 0.13, 0.08 and 0.07), while for the NTRK1 gene, no statistically significant differences were found (pFDR = 0.26; Figure 3). The most pronounced changes were observed for BDNF: the median relative expression decreased from 1.1 in the control group to 0.2 in the group of patients with depressive–anxiety disorders, while the type 2 diabetes group occupied an intermediate position.

When classifying samples using a conventional two-fold decrease in expression threshold, it was found that reduced BDNF expression was detected in 52% of participants with depressive–anxiety disorders and in 32% of patients with type 2 diabetes and 14% of patients with prediabetes, compared with 6% in the control group (χ^2^, *p* < 0.001; Cramer’s V = 0.33). Sensitivity analyses using 1.5× and 3× thresholds produced consistent between-group patterns (Supplementary Table S4). The conventional two-fold change threshold was chosen a priori as a widely used cutoff for biologically meaningful expression changes in RT-qPCR studies.

At the same time, NTRK1 expression did not differ between groups, suggesting that the observed differences were selective for BDNF- and NTRK2-related transcription rather than reflecting a uniform alteration of all measured neurotrophin-related genes. Because protein abundance, receptor activation and downstream signalling were not assessed, these findings should not be interpreted as direct evidence of altered BDNF–TrkB signalling activity.

Importantly, the participant-level analyses should not be interpreted as demonstrating that psychiatric and metabolic effects are fully separable. Rather, they quantify the relative statistical associations of measured affective and metabolic dimensions within an inherently overlapping phenotype structure. The persistence of the BDNF–PHQ-9 association after exclusion of the formally diagnosed depressive–anxiety group provides additional evidence that the observed relationship was not solely attributable to the categorical definition of that group.

### 3.3. Neurotrophins Track Mood and Vitamin D, Not Glycaemia

To assess the place of neurotrophins in the overall structure of metabolic and psychoemotional relationships, Spearman correlation analysis was performed in the entire study cohort. After correction using the Benjamini–Hochberg method, 52 out of 136 pairwise correlations (38.2%) retained statistical significance. A moderate inverse relationship was established between BDNF expression and the severity of depressive symptoms on the PHQ-9 scale (ρ = −0.49), as well as a moderate direct relationship between BDNF expression and the level of 25(OH)D (ρ = +0.47). Similar, although less pronounced, patterns were observed for NGF and NTRK2. At the same time, neurotrophin expression was practically not correlated with indicators of carbohydrate metabolism and obesity (|ρ| ≤ 0.16 for HbA1c, HOMA-IR and BMI), despite the close relationships between the metabolic indicators themselves. The strongest association with depressive symptom severity was demonstrated by 25(OH)D levels (ρ = −0.70), consistent with its potential regulatory role on BDNF expression.

Given the relationship between 25(OH)D levels and BDNF expression, it was analysed whether the association between BDNF and depressive symptomatology was explained solely by vitamin D status. Partial Spearman correlation analysis adjusted for 25(OH)D levels showed that the association between BDNF and PHQ-9 scores remained statistically significant (partial ρ = −0.25; *p* = 3 × 10^−4^). A similar result was obtained for the TrkB (NTRK2) receptor (partial ρ = −0.15; *p* = 0.03). After additional adjustment for BMI and age, the association between BDNF and PHQ-9 remained even stronger (partial ρ = −0.52; 95% CI −0.61 to −0.41; *p* < 0.001). In contrast, the partial correlations between HbA1c and PHQ-9 (ρ = −0.12; *p* = 0.085) and between BDNF and HbA1c (ρ = 0.05; *p* = 0.508) did not reach statistical significance.

In a multiple linear regression (OLS) model that included neurotrophins, 25(OH)D levels, and metabolic parameters (adjusted R^2^ = 0.65), 25(OH)D concentration showed the strongest statistical association with depressive symptom severity (standardised β = −0.68; 95% CI −0.79 to −0.57; *p* < 0.001, HC3 standard errors). On the raw scale, this corresponds to a decrease of approximately 5.1 PHQ-9 points per one standard deviation increase in 25(OH)D.

### 3.4. Participant-Level Heterogeneity Preserves Metabolic Signatures but Reveals Variable Neurotrophin Suppression

**Figure 4:**
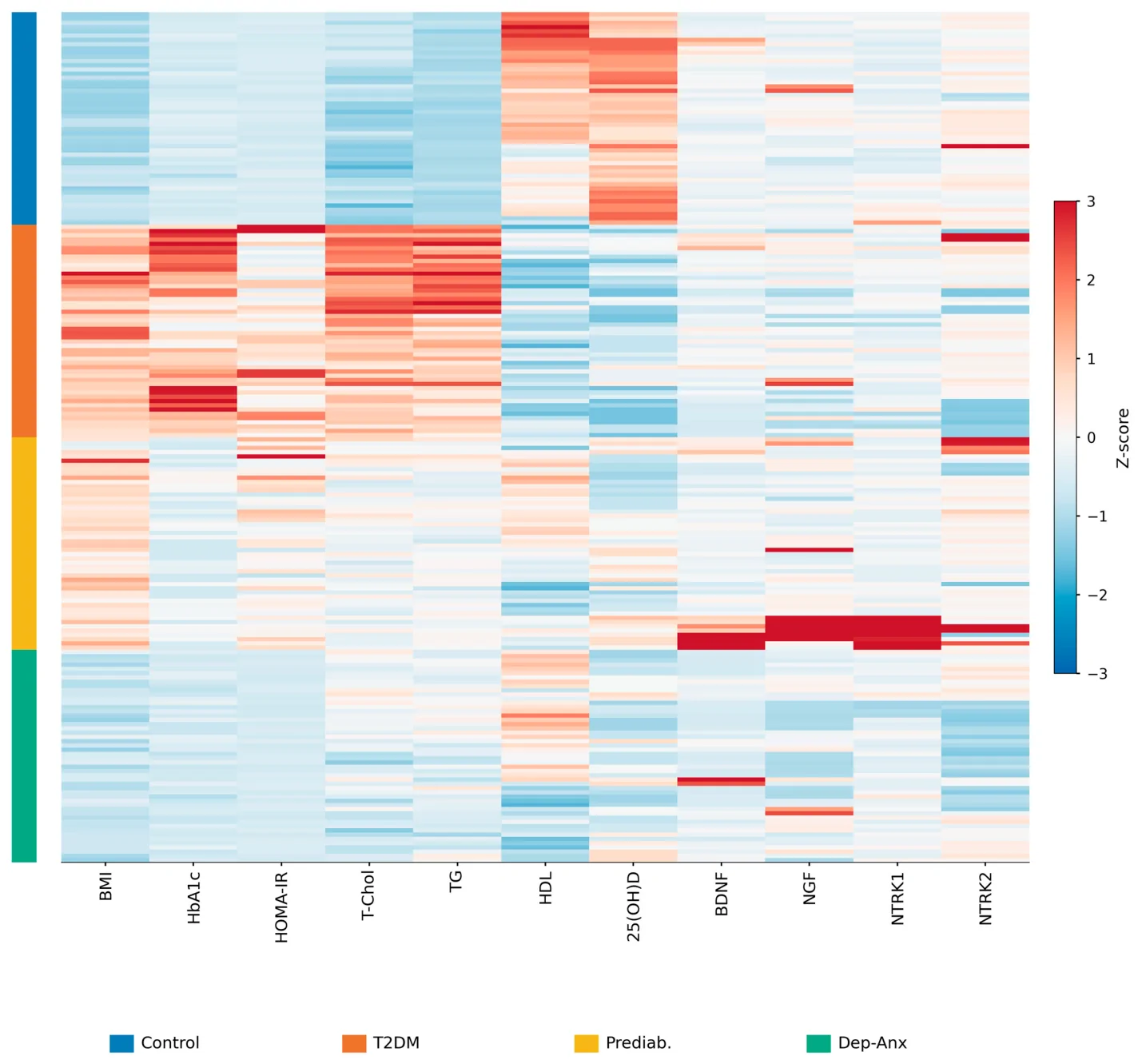
Standardised participant-level metabolic and gene-expression profiles. Row-standardised (z-score) values of the 14 metabolic and 4 neurotrophin/receptor gene-expression variables are shown for every participant (*n* = 200), grouped by clinical phenotype and hierarchically clustered (Ward linkage on Euclidean distance) within group. Colour scale is symmetric around the cohort mean. Metabolic signatures are tightly preserved within groups, whereas the neurotrophin columns—particularly BDNF and NTRK2 in the depressive–anxiety group—display marked inter-individual heterogeneity.

To move beyond group-level summaries, we z-standardised every metabolic and gene-expression variable within the cohort and displayed the individual profile for each of the 200 participants, hierarchically clustered within group (Figure 4). Two features are immediately apparent. First, metabolic characteristics were quite homogeneous within each study group. Participants with type 2 diabetes and prediabetes were characterised by consistently elevated rates of obesity, carbohydrate metabolism disorders, and dyslipidaemia, while in the group with depressive–anxiety disorders, the metabolic profile remained mostly normal and was similar to the control group.

Second, neurotrophin expression parameters showed significantly greater interindividual variability, especially among patients with depressive–anxiety disorders. Although a decrease in BDNF and NTRK2 expression was characteristic of the majority of participants in this group, in a significant part of the examined subjects, the level of expression remained close to control values.

The observed interindividual heterogeneity is consistent with moderate but statistically significant intergroup differences for BDNF, NGF, and NTRK2 (Table 1). The results obtained indicate that changes in neurotrophin expression are not universal for all patients, but reflect individual biological characteristics, which justifies the need to use multivariate statistical analysis to identify factors associated with the suppression of their expression.

### 3.5. Neurotrophins Decline Progressively with Psychiatric Severity

**Figure 5:**
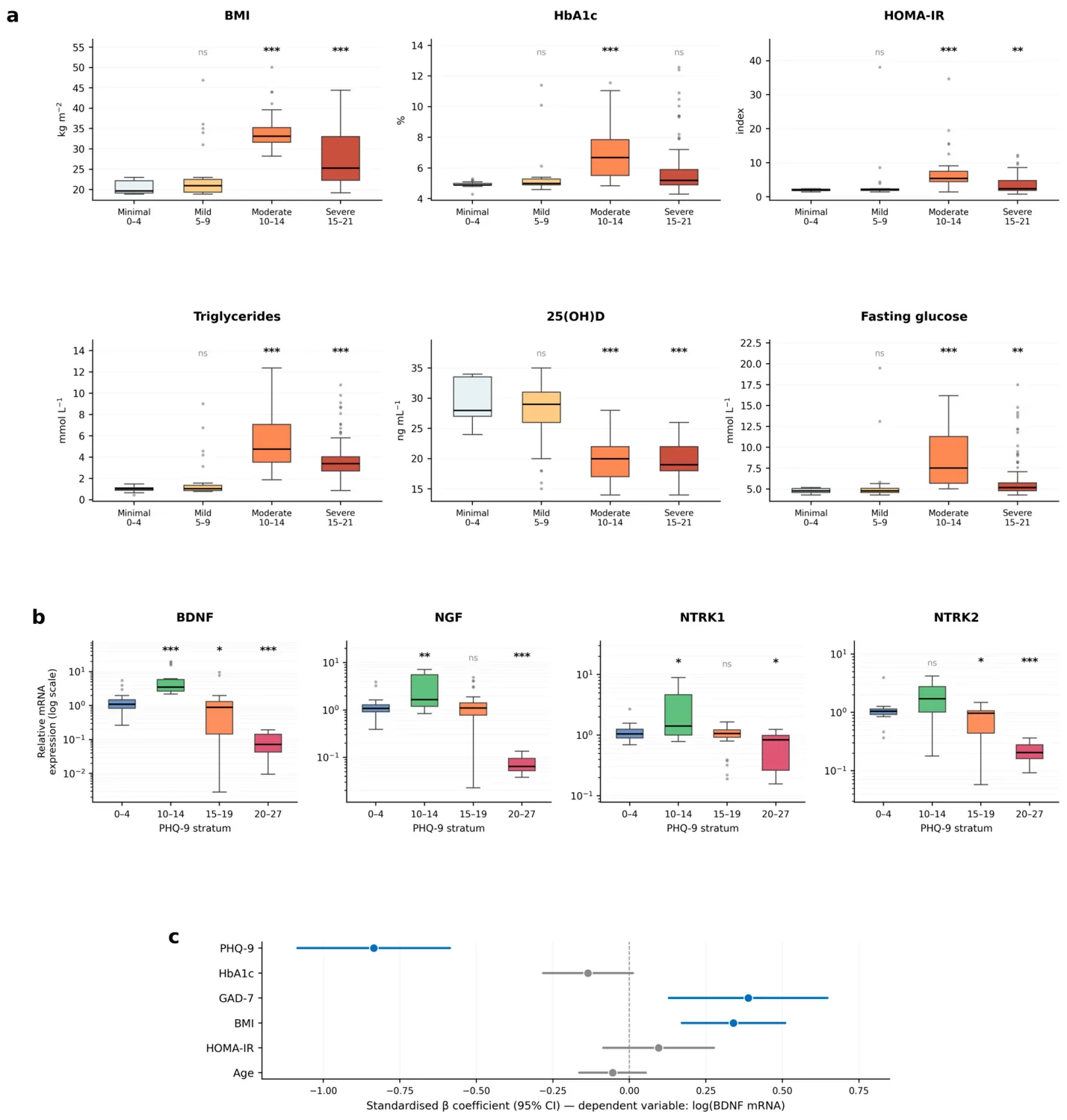
Psychiatric severity gradients and multivariable predictors of peripheral BDNF expression. (**a**) Distribution of six metabolic parameters (BMI, HbA1c, HOMA-IR, triglycerides, 25(OH)D, fasting glucose) across GAD-7 anxiety severity strata (minimal, mild, moderate, severe), pooling all four clinical groups (*n* = 200). Boxes show median and IQR; whiskers 1.5 × IQR. (**b**) Relative peripheral expression (log scale, RT-qPCR, control = 1.0) of BDNF, NGF, NTRK1 and NTRK2 across PHQ-9 depression severity strata (0–4/5–9/10–14/15–19/20–27), pooling all four groups. BDNF, NGF and NTRK2 decline monotonically with severity; NTRK1 is invariant. (**c**) Forest plot of standardised regression coefficients (β with 95% CI, HC3-robust) from the multivariable OLS model of log(BDNF) on PHQ-9, GAD-7, BMI, HbA1c, HOMA-IR and age (apparent R^2^ = 0.257, *n* = 200). Filled points denote *p* < 0.05; open points, *p* ≥ 0.05. PHQ-9 showed the strongest statistical association with log(BDNF); the positive GAD-7 coefficient reflects a suppression effect from collinearity with PHQ-9 (all VIF < 5). Robust (Hube^r^ M) and median (τ = 0.5) regression estimates converge on the same conclusions and are reported in the text. * indicates *p* < 0.05; ** indicates *p* < 0.01; *** indicates *p* < 0.001; ns indicates no statistically significant difference (*p* ≥ 0.05).

Pooling all four groups and stratifying by PHQ-9 depression and GAD-7 anxiety severity revealed a coherent graded pattern across the psychiatric spectrum (Figure 5a,b). Metabolic parameters (BMI, HbA1c, HOMA-IR, triglycerides, 25(OH)D, fasting glucose) shifted monotonically across GAD-7 severity strata (Figure 5a), and BDNF, NGF and NTRK2 expression declined progressively from minimal to very-pronounced PHQ-9 severity, whereas NTRK1 remained flat (Figure 5b). Because PHQ-9 and GAD-7 are tightly co-varying in this cohort (ρ = 0.77), the two gradients index a shared psychiatric symptom axis rather than clearly separable statistical associations. A progressive reduction in BDNF and NTRK2 transcript levels was observed across increasing affective symptom burden, whereas NTRK1 expression remained comparatively stable. Because these measurements were obtained from whole blood and were limited to mRNA expression, the pattern should be interpreted as an association between affective symptom burden and peripheral BDNF/NTRK2-related transcription rather than as evidence of receptor-level signalling changes or a causal effect of psychiatric status.

### 3.6. Multivariable Modelling Identifies PHQ-9, GAD-7 and BMI as Statistical Correlates of Log(BDNF)

To quantify the relative statistical contribution of each domain, we regressed log(BDNF) on both the psychiatric block (PHQ-9, GAD-7) and the metabolic block (BMI, HbA1c, HOMA-IR) with age as a covariate (apparent R^2^ = 0.257; Table 2, Figure 5b,c). In the multivariable OLS model, PHQ-9 showed the strongest statistical association with log(BDNF), while BMI and GAD-7 also showed statistically significant associations; HbA1c, HOMA-IR and age were not statistically significant (Table 2). Robust and median regression produced the same direction of association for PHQ-9, while the HOMA-IR coefficient was statistically equivalent to a negligible association in the TOST analysis.

### 3.7. Bootstrap Decomposition of Associations and Correlation Network Position BDNF Closer to the Psychiatric than the Metabolic Domain

**Figure 6:**
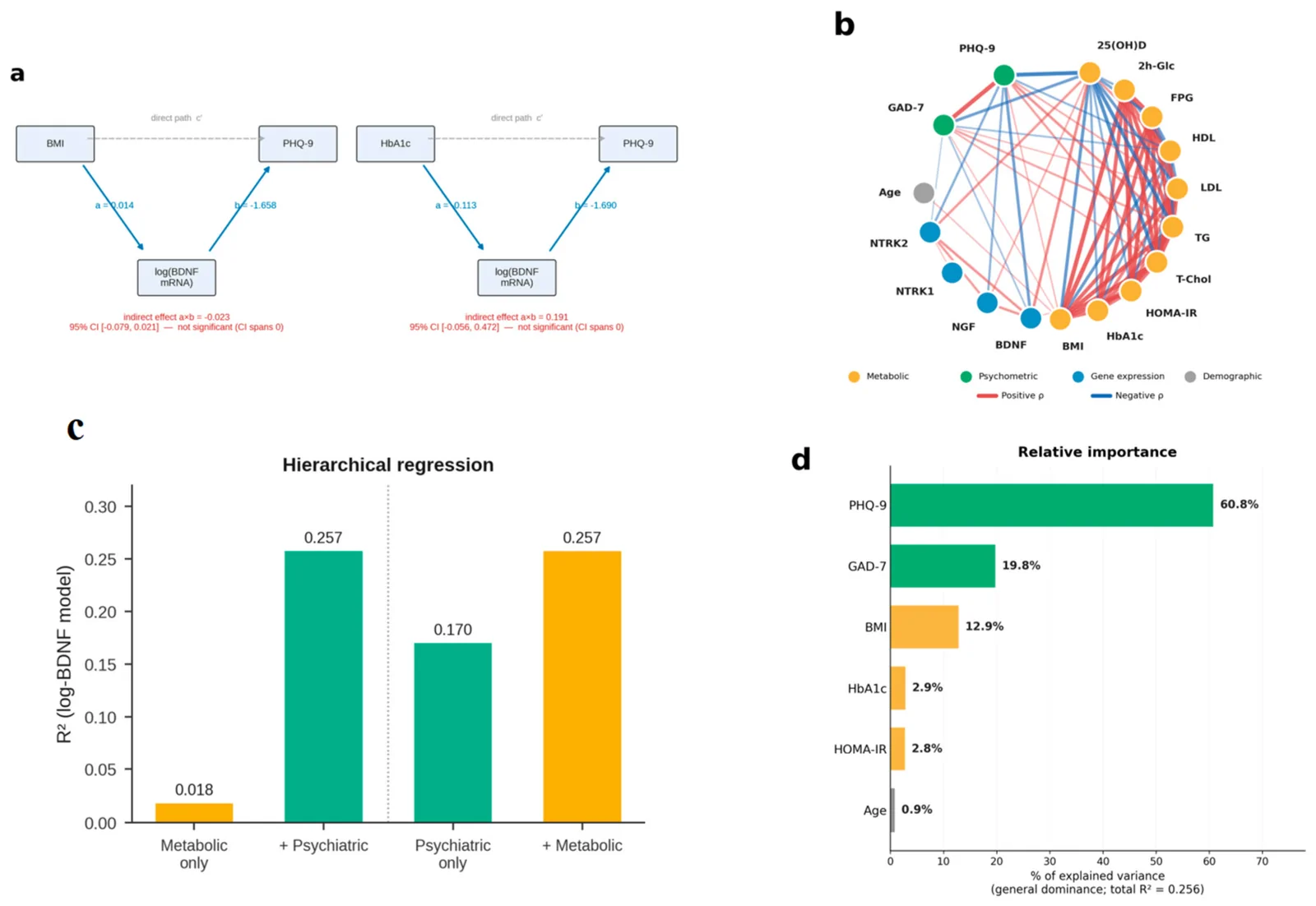
Statistical architecture of the psychiatric–metabolic contribution to peripheral BDNF variance. (**a**) Bootstrap statistical decomposition path diagrams (5000 resamples) for BMI → log(BDNF) → PHQ-9 (left) and HbA1c → log(BDNF) → PHQ-9 (right), with age (and, for the HbA1c model, BMI) as covariates. Neither indirect effect reached significance (95% CI spans zero). (**b**) Weighted correlation network across all metabolic (yellow), psychometric (green), gene-expression (blue) and demographic (grey) variables. Edges represent Benjamini–Hochberg-significant Spearman correlations (52 of 136 pairs; adjusted *p* < 0.05); edge width scales with |ρ| and colour indicates sign (red—positive, blue—negative). BDNF, NGF and NTRK2 cluster closer to the psychometric than to the metabolic domain. (**c**) Hierarchical regression on log(BDNF): R^2^ when the psychiatric block (PHQ-9, GAD-7) is added to a metabolic + age base model versus when the metabolic block (BMI, HbA1c, HOMA-IR) is added to a psychiatric + age base model. (**d**) General dominance analysis: predictor-specific contributions to the total explained variance of log(BDNF) (apparent R^2^ = 0.257), computed as the mean marginal R^2^ across all 2^6^ = 64 subsets of the six predictors and normalised to sum to 100%. Psychiatric variables account for 80.6% of explained variance, metabolic variables 18.6%, and age 0.9%. All statistics: *n* = 200; two-sided; *p*_FDR_ where applicable. Cross-sectional design; no causal claims implied.

The next step was to statistically decompose the association between metabolic characteristics and depressive symptom severity into a component statistically associated with peripheral BDNF expression and a residual association. This decomposition should be interpreted as an exploratory partition of statistical associations rather than as a causal mediation analysis, given the cross-sectional design. We performed a bootstrap decomposition of the indirect association (5000 replicates), treating log(BDNF) as the intermediate variable in the BMI → PHQ-9 relationship, with age as a covariate. The resulting indirect effect was not statistically significant (a × b = −0.023; 95% CI −0.079 to +0.021; Figure 6a). A similar analysis for the HbA1c → PHQ-9 model (adjusted for BMI and age) also did not reveal a statistically significant indirect effect (a × b = +0.191; 95% CI −0.056 to +0.472).

In both models, the weak relationship between metabolic parameters and BDNF expression (path a) limited the magnitude of the indirect effect, despite the presence of a pronounced association between BDNF expression and depressive symptom severity. The results obtained indicate the lack of statistical evidence that the observed association between metabolic characteristics and depressive symptoms is statistically mediated by a decrease in peripheral BDNF expression. Instead, the findings are compatible with the possibility that BDNF expression and depressive symptom severity are influenced by shared upstream factors, although such mechanisms cannot be established from the present cross-sectional data.

These conclusions are supported by the results of the weighted correlation network analysis (Figure 6b), in which BDNF, NGF and NTRK2 were spatially closer to the psychometric parameters than to the group of metabolic variables. This structure is consistent with the results of the partial correlation and regression analyses and indicates a closer relationship of neurotrophins with psychoemotional characteristics than with indicators of metabolic status. At the same time, it should be noted that the results presented reflect only statistical relationships and do not allow conclusions to be drawn about cause-and-effect relationships, since the study has a cross-sectional design.

### 3.8. Variance Partitioning: Psychiatric Symptom Burden Accounts for Most of the Modelled Variance in BDNF, While BMI Modified the Observed Association

Hierarchical regression analysis of log-transformed BDNF expression demonstrated significant differences in the contribution of metabolic and psychiatric variables (Table 3; Figure 6c,d). The metabolic-only model had an apparent R^2^ of 0.018 (1.8% of the variance in log(BDNF)), whereas the full model including psychiatric variables had an apparent R^2^ of 0.257 (25.7% of the variance). The incremental contribution of psychiatric variables was larger than that of the metabolic block in both hierarchical entry orders (Table 3). Dominance analysis attributed 80.6% of the model’s explained variance to the psychiatric block, compared with 18.6% to metabolic variables and 0.9% to age. Bootstrap internal validation (1000 resamples, Harrell’s method) yielded a bootstrap optimism-corrected R^2^ of 0.213 (optimism = 0.044). The PHQ-9 × BMI interaction indicated that BMI modified the observed association between depressive symptom severity and BDNF expression.

Taken together, the participant-level analyses provide a more specific biological interpretation than the between-group comparisons alone. Although BDNF, NGF and NTRK2 expression differed across the four clinical phenotypes, the variance-partitioning analyses showed that psychiatric symptom burden accounted for 80.6% of the explanatory contribution to the multivariable model, compared with 18.6% for the metabolic variables and 0.9% for age. The association of BDNF with depressive symptom severity remained significant after adjustment for 25(OH)D, BMI, HbA1c, HOMA-IR and age, whereas HbA1c and HOMA-IR showed no significant independent association with BDNF. Moreover, the significant PHQ-9 × BMI interaction indicated that BMI modified the observed association between depressive symptoms and BDNF expression. These findings should also be interpreted in the context of the ongoing war in Ukraine, which represents an important population-level source of chronic psychological stress and may contribute to the substantial psychiatric symptom burden observed in the study population. Although war-related exposure was not directly quantified in the present analyses, its potential contribution to affective symptom severity should be considered when interpreting the predominance of psychiatric variables in the variance-partitioning model. Thus, the principal signal identified in this study was a preferential alignment of peripheral BDNF transcription with affective symptom burden, with metabolic status acting mainly as a modifying context rather than the primary statistical correlate, within a broader context of sustained war-related stress.

## 4. Discussion

In a cohort comprising four clinically distinct groups, we observed differences in peripheral BDNF and NTRK2 mRNA expression across the metabolic–affective spectrum, with the lowest values occurring in participants with the greatest affective symptom burden. The concurrent reduction in BDNF and NTRK2 transcript levels, together with relatively unchanged NTRK1 expression, suggests a selective pattern of peripheral neurotrophin-related transcriptional changes rather than a uniform alteration across all measured genes. However, because protein abundance, receptor phosphorylation and downstream signalling were not measured, these results do not demonstrate altered BDNF–TrkB signalling activity [26,27,28,67,68,69].

The main additional insight provided by this study is therefore not the identification of another association between BDNF and depression or between vitamin D and affective symptoms. Instead, the data provide evidence regarding the relative positioning of peripheral neurotrophin transcription within overlapping affective and metabolic domains. Across the same participants, depressive symptom burden showed a substantially larger contribution to the explained variance in BDNF than the metabolic block, while HbA1c and HOMA-IR were not independently associated with BDNF. This pattern remained evident after adjustment for vitamin D status and other metabolic characteristics and was supported by robust and median regression analyses. At the same time, the significant PHQ-9 × BMI interaction suggests that adiposity may modify the strength of the association between affective symptoms and peripheral BDNF, rather than acting as its primary driver. Importantly, these findings should be considered within the broader context of the ongoing war in Ukraine, where prolonged exposure to uncertainty, chronic stress, displacement, loss, and other war-related stressors may contribute to the overall affective symptom burden of the population. Although war-related exposure was not directly quantified in the present study and therefore cannot be evaluated as an independent determinant of BDNF transcription, it represents a relevant contextual factor when interpreting the predominance of affective symptoms in the observed associations. Thus, the findings suggest that, within this particular cross-sectional cohort exposed to a broader context of sustained war-related stress, peripheral whole-blood BDNF transcription is more closely aligned with affective symptom burden than with the degree of glycaemic dysregulation.

The most important clinical result was the discovery of a close relationship between vitamin D levels, the severity of depressive symptoms and the expression of neurotrophins. Vitamin D showed the strongest statistical association with depression severity and was the variable most strongly associated with clinically significant depression in the present model, consistent with its role as a transcriptional regulator of BDNF [42,43,44] and its independent association with mood disorders [45,46,70]. However, the association between BDNF and depression persisted after adjustment for vitamin D levels, indicating that this association was not fully accounted for by vitamin D status.

Neurotrophin expression was not significantly associated with glycaemic control. Despite the known role of BDNF in energy metabolism [34,36,37,70], in our study, its expression was more closely associated with affective symptoms and vitamin D status than with HbA1c, HOMA-IR, or BMI. This suggests that peripheral BDNF suppression is primarily a marker of the psychoemotional changes that accompany metabolic diseases, rather than a direct consequence of them [33,71,72,73,74].

The absence of a significant association between peripheral BDNF expression and glycaemic control in the present cohort should be interpreted in the context of the heterogeneous findings reported previously. Experimental and clinical studies have suggested a role for BDNF in glucose and energy metabolism, including evidence linking BDNF with type 2 diabetes and obesity-related metabolic phenotypes [49,75,76,77,78,79]. However, other studies have reported increased rather than decreased circulating BDNF in specific metabolic contexts, particularly in newly diagnosed patients with type 2 diabetes and obesity [36], indicating that the relationship between BDNF and metabolic status may depend on disease stage, adiposity, sex, and the biological compartment examined. An important distinction is that the present study quantified BDNF mRNA expression in whole peripheral blood, whereas many previous investigations assessed circulating serum or plasma BDNF protein concentrations. These measures reflect different biological levels and cannot be assumed to show identical associations with metabolic variables. In addition, our multivariable analyses simultaneously accounted for affective symptom burden, adiposity and insulin resistance, allowing the psychiatric and metabolic contributions to BDNF transcription to be compared within the same analytical framework. Under these conditions, HbA1c and HOMA-IR showed weak associations with BDNF expression, whereas depressive symptom severity showed the strongest statistical association. Thus, the present findings do not exclude a metabolic role of BDNF; rather, they suggest that, in this cohort, variation in whole-blood BDNF transcription was more closely related to affective symptom burden and vitamin D status than to the glycaemic indices examined. Differences in study populations, disease stage, assay type, tissue or circulating compartment, and adjustment strategies may therefore contribute to the apparently conflicting results across studies.

Additional analyses confirmed these results. Hierarchical regression showed that psychiatric indicators explained approximately 2.7 times more of the variability in log(BDNF) levels than metabolic factors (ΔR^2^ = 0.24 vs. 0.09), and dominance analysis showed that approximately 81% of the variance explained by the model (apparent R^2^ = 0.257) was accounted for by PHQ-9 and GAD-7, which corresponds to approximately 20.7% of the total observed variance in log(BDNF) expression. BMI, HbA1c, and HOMA-IR jointly contributed approximately 19% of the explained variance (i.e., ~4.9% of the total variance) (Table 3, Figure 6c,d). Exploratory mediation decomposition did not provide statistical evidence that BDNF accounted for any part of the associations between BMI or HbA1c and depression severity. The correlation network also showed a stronger association of BDNF, NGF, and NTRK2 with psychometric measures than with metabolic measures (Figure 6a,b). The PHQ-9 × BMI interaction indicated that BMI modified the observed association between depressive symptoms and BDNF expression, which is consistent with data on the role of chronic inflammation in obesity [12,80,81,82,83]. At the same time, these results are consistent with a strong association of 25(OH)D with peripheral BDNF expression and depressive symptom severity, but do not by themselves establish a regulatory axis. It should be noted that the results of the dominance analysis and statistical decomposition reflect only statistical relationships and do not allow for causal conclusions.

A biomarker panel combining peripheral log(BDNF) and log(NTRK2) expression with serum 25(OH)D showed strong in-sample discrimination of PHQ-9 ≥ 15 (5-fold cross-validated AUC 0.97, 95% CI 0.95–0.98; sensitivity 0.92, specificity 0.84; only marginal improvement over 25(OH)D alone: AUC 0.95, 95% CI 0.92–0.97; Supplementary Table S5). However, participants in the depressive–anxiety group were selected on the basis of psychiatric symptom severity, so this estimate is likely optimistically biased. We therefore report the classification analysis as exploratory. External validation in an independent cohort using outcomes defined independently of the selection procedure is required before any claim of clinical utility can be made.

Sensitivity analyses supported the robustness of the primary findings (Supplementary Table S3). First, restricting the multivariable model to participants without a formal psychiatric diagnosis (i.e., excluding the depressive–anxiety group) preserved the negative association between BDNF and PHQ-9 (β = −0.14; 95% CI −0.24 to −0.04; *p* = 0.008). Second, adjustment for sex and for use of antidepressants, metformin, statins and vitamin D supplementation did not materially change the coefficients of interest (Supplementary Table S3). Third, seasonal stratification of 25(OH)D measurements (May–October vs November–April) preserved the direction and significance of its association with PHQ-9. Fourth, correlations examined separately within each clinical group showed consistent directionality for BDNF–PHQ-9 and 25(OH)D–PHQ-9, although magnitudes were attenuated within groups, consistent with the between-group contrasts contributing to the pooled correlations. The complete analytical code is provided as File S3. Importantly, we do not interpret the participant-level analyses as removing the fundamental overlap between psychiatric and metabolic phenotypes. Rather, they provide a way to quantify associations within that overlap.

These findings should be positioned carefully within the existing literature. Meta-analyses of circulating BDNF protein have reported reduced levels in both major depressive disorder and type 2 diabetes, but effect sizes are heterogeneous and are attenuated by treatment, comorbidities and assay method. Our data are consistent with these reports at the mRNA level and add that, when affective and metabolic dimensions are examined jointly, the neurotrophin signal aligns more closely with psychiatric than with glycaemic burden. Several alternative explanations should nevertheless be considered. First, the observed BDNF and NTRK2 patterns could reflect shared upstream drivers—chronic low-grade inflammation, HPA-axis activation, oxidative stress or adiposity—rather than a specific vitamin D–neurotrophin relationship. Second, antidepressant treatment itself modulates peripheral BDNF, and although sensitivity analyses (Supplementary Table S3) did not change the direction of the effect, residual confounding cannot be excluded. Third, PHQ-9 and GAD-7 are symptom-severity instruments and do not by themselves establish clinical diagnoses in participants outside the depressive–anxiety group; some misclassification of affective burden across the metabolic groups is therefore inevitable. These considerations frame the results as an exploratory biomarker association rather than a validated clinical tool.

The study has several limitations that qualify the interpretation of our findings. (i) Cross-sectional design. All variables were measured at a single time point; temporal ordering and causal direction cannot be established, and any causal or mechanistic language throughout the manuscript should be read as association-based. (ii) Peripheral neurotrophin assessment. Neurotrophin expression was measured in whole peripheral blood, which reflects a mixture of transcripts from mononuclear cells, granulocytes and other components; peripheral mRNA correlates only partially with central nervous system BDNF/NTRK2 activity, and differential leukocyte composition—which we did not quantify by flow cytometry or CBC differential—may have contributed to the between-group differences observed. (iii) Overlap between affective and metabolic phenotypes. The four clinical groups were designed to provide clinically contrasting phenotypes but were not based on a factorial design in which psychiatric and metabolic status were independently assigned. Consequently, affective symptoms were also present among participants with prediabetes and type 2 diabetes, and the depressive–anxiety group was not completely isolated from all biological factors that may influence neurotrophin expression. Participant-level regression, partial correlation and variance-partitioning analyses quantify the relative associations of the measured psychiatric and metabolic dimensions but cannot remove unmeasured confounding or transform the observational design into independent causal contrasts. The findings should therefore be interpreted as evidence for differential statistical alignment of BDNF expression with affective versus metabolic burden, rather than as definitive separation of psychiatric and metabolic effects. (iv) Medication and lifestyle confounding. Although the use of antidepressants, metformin, statins and vitamin D supplementation was recorded and entered in sensitivity models (Supplementary Table S3), residual confounding by dose, duration, adherence, diet, physical activity and sleep cannot be excluded [12,80,84,85,86,87,88]. (v) Systemic inflammation. hs-CRP, IL-6 and other inflammatory markers were not measured; low-grade inflammation is a plausible shared contributor to reduced BDNF, low 25(OH)D and depressive symptoms, and its role cannot be disentangled from the present data. (vi) Single-centre recruitment. The cohort was drawn from a single Ukrainian regional centre, which limits generalisability to other populations, latitudes and health-system contexts.

### Take-Home Message and Future Research 

In an integrated four-group cohort spanning a prediabetes–T2DM–depressive–anxiety spectrum, peripheral BDNF and NTRK2 mRNA and serum 25(OH)D tracked depressive symptom burden more closely than glycaemic or anthropometric variables, and the psychiatric contribution dominated the explained variance of a multivariable model in which both dimensions competed. These findings identify a candidate peripheral biomarker pattern rather than a validated clinical tool. Priority next steps are: (i) prospective replication with baseline and follow-up measurements to establish temporal ordering; (ii) parallel assessment of BDNF and NTRK2 at the protein level, together with inflammatory markers (hs-CRP, IL-6) and leukocyte composition, to test whether transcript changes reflect biologically active signalling; (iii) interventional studies of vitamin D repletion in depressive–anxiety phenotypes with dysglycaemia; and (iv) external validation of the BDNF + NTRK2 + 25(OH)D panel in independent cohorts where the outcome is defined independently of the biomarker set.

## 5. Conclusions

In this cross-sectional cohort, peripheral BDNF and NTRK2 mRNA expression differed across the predefined metabolic and affective phenotypes and showed stronger statistical associations with depressive symptom severity and 25(OH)D status than with the examined measures of glycaemic control. The association between BDNF expression and depressive symptoms remained significant after adjustment for 25(OH)D, BMI, HbA1c, HOMA-IR, and age. Variance-partitioning and dominance analyses indicated a greater contribution of psychiatric variables than metabolic variables to the explained variability in BDNF expression. The PHQ-9 × BMI interaction further indicated that BMI modified the observed association between depressive symptoms and BDNF expression. These findings should be interpreted in the context of the ongoing war in Ukraine, which may contribute to the affective symptom burden in the study population, although war-related exposure was not directly assessed.

These findings describe peripheral transcriptional associations and do not establish altered BDNF–TrkB signalling, causality, or clinical utility. Prospective and interventional studies incorporating protein-level measurements and downstream signalling markers are required to determine the biological and clinical significance of these associations.

## Figures and Tables

**Table 1:** Baseline characteristics by clinical group, median (interquartile range).

Variable	Control (*n* = 50)	Prediabetes (*n* = 50)	T2DM (*n* = 50)	Dep-Anx (*n* = 50)	*p* Value
Age, years	41.0 (37.0–46.8)	41.0 (33.0–47.0)	42.0 (39.0–44.8)	38.0 (32.0–44.0)	0.132
Sex, female n (%)	30 (60.0%)	30 (60.0%)	26 (52.0%)	37 (74.0%)	0.076
BMI, kg/m^2^	19.9 (19.3–22.0)	31.9 (30.4–33.4)	34.5 (33.0–37.0)	22.4 (21.3–24.0)	<0.001
Weight, kg	66.0 (66.0–69.5)	88.0 (82.0–92.0)	93.5 (82.0–114.5)	74.0 (66.2–76.0)	<0.001
HbA1c, %	5.0 (4.9–5.1)	5.5 (4.9–5.9)	8.0 (7.2–9.7)	4.9 (4.7–5.2)	<0.001
HOMA-IR	2.0 (1.8–2.2)	4.8 (4.1–6.8)	6.4 (4.4–8.1)	1.9 (1.7–2.2)	<0.001
Total chol., mmol/L	4.48 (4.06–4.90)	6.06 (5.75–6.47)	8.66 (7.86–9.43)	5.71 (5.31–6.12)	<0.001
LDL-C, mmol/L	2.51 (2.29–2.94)	3.44 (3.09–3.71)	4.69 (4.05–5.23)	3.25 (2.78–3.56)	<0.001
HDL-C, mmol/L	1.47 (1.27–1.61)	1.18 (1.03–1.29)	0.92 (0.82–1.05)	1.19 (0.93–1.40)	<0.001
Triglycerides, mmol/L	1.00 (0.86–1.14)	3.51 (3.04–3.79)	6.49 (5.47–8.69)	2.76 (2.35–3.44)	<0.001
Fasting gluc, mmol/L	4.8 (4.6–5.1)	5.7 (5.6–5.8)	10.3 (8.2–13.5)	4.8 (4.6–5.0)	<0.001
2-h glucose, mmol/L	5.9 (5.7–6.1)	8.0 (7.6–8.6)	10.7 (9.5–14.0)	5.7 (5.5–5.9)	<0.001
25(OH)D, ng/mL	29.0 (27.0–32.8)	22.0 (18.0–24.0)	18.0 (15.0–20.0)	19.0 (18.0–22.0)	<0.001
PHQ-9 score	4.0 (3.0–4.0)	16.0 (15.0–17.0)	17.0 (16.0–17.0)	19.0 (18.0–19.0)	<0.001
GAD-7 score	5.0 (4.0–5.8)	17.0 (11.0–18.0)	13.0 (12.0–16.0)	19.0 (18.0–20.0)	<0.001
BDNF, fold	1.1 (0.8–1.5)	1.3 (0.9–1.9)	1.0 (0.1–1.5)	0.2 (0.1–1.0)	<0.001
NGF, fold	1.1 (0.9–1.3)	1.3 (0.9–1.5)	1.0 (0.7–1.4)	0.9 (0.1–1.3)	<0.001
NTRK1, fold	1.1 (0.9–1.2)	1.1 (0.9–1.3)	1.1 (0.9–1.2)	1.0 (0.9–1.2)	0.261
NTRK2, fold	1.0 (0.9–1.1)	1.0 (1.0–1.1)	1.0 (0.2–1.1)	0.8 (0.3–1.0)	<0.001

*p* values from Kruskal–Wallis omnibus tests; all metabolic and psychometric comparisons survive Benjamini–Hochberg correction. BMI, body-mass index; HOMA-IR, homeostatic model assessment of insulin resistance; LDL-C/HDL-C, low-/high-density-lipoprotein cholesterol; 25(OH)D, 25-hydroxyvitamin D; PHQ-9, Patient Health Questionnaire-9; GAD-7, Generalised Anxiety Disorder-7. Neurotrophins expressed as relative fold-change versus the healthy reference. Additional variables (sex distribution, smoking status, alcohol use, education, fasting insulin, WHO-5, FINDRISC, PAID, blood-collection season, medication and supplement use) are reported in Supplementary Table S2.

**Table 2:** Multivariable regression models of log(*BDNF*) and related outcomes (standardised β, 95% CI, *p*).

Model/Term	β	95% CI	*p*
**OLS: log(** ***BDNF*** **) ~ PHQ-9 + GAD-7 + BMI + HbA1c + HOMA-IR + age (apparent R^2^ = 0.257; ** ***n*** ** = 200)**			
PHQ-9	−0.207	−0.270, −0.144	<0.001
GAD-7	+0.106	+0.040, +0.171	0.002
BMI	+0.075	+0.037, +0.113	<0.001
HbA1c	−0.121	−0.264, +0.021	0.094
HOMA-IR	+0.034	−0.017, +0.085	0.196
Age	−0.012	−0.037, +0.014	0.373
**Robust (Huber M-estimation): log(** ***BDNF*** **) ~ same predictors**			
PHQ-9	−0.183	−0.239, −0.127	<0.001
HbA1c	–	–	0.253
**Median (quantile τ = 0.5) regression: log(** ***BDNF*** **) ~ same predictors**			
PHQ-9	−0.149	−0.202, −0.096	<0.001
HbA1c	≈0	–	0.977
**OLS: HbA1c ~ PHQ-9 + GAD-7 + BMI + age (apparent R^2^ = 0.381)**			
BMI	+0.145	+0.115, +0.176	<0.001
PHQ-9	+0.040	−0.022, +0.103	0.205
GAD-7	−0.061	−0.125, +0.004	0.067
**Ordinal logistic: PHQ-9 severity ~ metabolic panel**			
HbA1c	−0.517	−0.803, −0.231	<0.001
25(OH)D	−0.750	−0.939, −0.562	<0.001
BMI	+0.006	−0.068, +0.080	0.875

All continuous predictors were z-standardised. HC3 standard errors used for OLS. All VIFs < 5. The positive GAD-7 coefficient in the log(BDNF) OLS model reflects a statistical suppression effect from collinearity with PHQ-9 (VIF 3.94–4.35) and represents the residual, PHQ-9-independent component of anxiety symptomatology rather than a positive biological effect. Two one-sided equivalence tests (TOST, ±0.15 margin) confirmed the HOMA-IR coefficient as statistically equivalent to a negligible effect (*p* < 0.001); HbA1c and age were directionally small but not conclusively equivalent (*p* = 0.346 and 0.261).

**Table 3:** Variance partitioning for log(*BDNF*): hierarchical regression, general dominance analysis and bootstrap internal validation.

Analysis	Metric	Value
Hierarchical regression	R^2^, metabolic block + age only	0.018
	R^2^, metabolic block + age + psychiatric block	0.257
	ΔR^2^ (psychiatric added second)	0.239 (F = 31.08, *p* = 1.99 × 10^−12^)
	R^2^, psychiatric block + age only	0.170
	R^2^, psychiatric block + age + metabolic block	0.257
	ΔR^2^ (metabolic added second)	0.088 (F = 7.58, *p* < 0.001)
General dominance (% of total apparent R^2^ = 0.257)	PHQ-9	60.8%
	GAD-7	19.8%
	BMI	12.9%
	HOMA-IR	2.8%
	HbA1c	2.9%
	Age	0.9%
	Psychiatric block total	80.6%
	Metabolic block total	18.6%
Bootstrap internal validation (1000 resamples)	Apparent R^2^	0.257
	Optimism	0.044
	Bootstrap optimism-corrected R^2^	0.213
PHQ-9 × BMI interaction	β for interaction term	−0.0157 (95% CI −0.026 to −0.005), *p* = 0.003
	Simple slope of PHQ-9 → log(*BDNF*) at BMI ≈ 20 kg m^−2^ (lean)	−0.151
	Simple slope at cohort-mean BMI (27.8 kg m^−2^)	−0.273
	Simple slope at BMI ≈ 34.6 kg m^−2^ (obese)	−0.381

Hierarchical regression F-statistics test the significance of the incremental R^2^ attributable to each block. General dominance percentages are Shapley-value decompositions of R^2^ averaged across all 2^6^ = 64 predictor orderings. Bootstrap optimism was estimated by Harrell’s method (1000 resamples). Simple slopes are evaluated at ±1 SD of BMI. Cross-sectional design; percentages describe relative explanatory contributions, not causal effects.

## Data Availability

The data presented in this study are available from the corresponding author upon reasonable request. The data are not publicly available because they contain information that could compromise the privacy of the research participants.

## Supplementary Materials

The following supporting information can be downloaded at: https://www.mdpi.com/article/10.3390/biomedicines14092106/s1, Table S1: Primer sequences and amplification characteristics for RT-qPCR. Table S2: Extended baseline characteristics of the study cohort by clinical group. Table S2b: Clinical characteristics of the depressive–anxiety group. Table S3: Sensitivity analyses for the primary association between depressive symptom severity and peripheral BDNF expression. Table S4: Reduced peripheral BDNF expression across alternative fold-change thresholds. Table S5: Exploratory classification of clinically significant depression (PHQ-9 ≥ 15). File S1: MIQE checklist for RT-qPCR reporting; File S2: STROBE checklist for cross-sectional studies; File S3: Executed Jupyter notebook reproducing every reported analysis (Python 3.12; .ipynb and .html render). Reference [89] is cited in the Supplementary Material.

## Abbreviations

The following abbreviations are used in this manuscript:

ACTB	Beta-actin
ADA	American Diabetes Association
BDNF	Brain-Derived Neurotrophic Factor
BMI	Body Mass Index
CI	Confidence Interval
GAD-7	Generalized Anxiety Disorder-7
HbA1c	Glycated Haemoglobin
HC3	Heteroscedasticity-Consistent Standard Errors (HC3)
HDL-C	High-Density Lipoprotein Cholesterol
HOMA-IR	Homeostatic Model Assessment of Insulin Resistance
LDL-C	Low-Density Lipoprotein Cholesterol
NGF	Nerve Growth Factor
OLS	Ordinary Least Squares
PAID	Problem Areas in Diabetes
PHQ-9	Patient Health Questionnaire-9
RT-qPCR	Reverse Transcription Quantitative Polymerase Chain Reaction
T2DM	Type 2 Diabetes Mellitus
TrkA	Tropomyosin Receptor Kinase A (NTRK1)
TrkB	Tropomyosin Receptor Kinase B (NTRK2)
WHO-5	World Health Organization Well-Being Index
25(OH)D	25-Hydroxyvitamin D
