# Supplementary material for: Peripheral BDNF–TrkB Expression and Vitamin D Status in Depression, Prediabetes and Type 2 Diabetes: A Cross-Sectional Biomarker Study
Source: Biomedicines. 2026 Sep 18;14(9):2106. doi: 10.3390/biomedicines14092106 (PMC13604075; doi:10.3390/biomedicines14092106)
Supplement: Supplementary file 1 [file biomedicines-14-02106-s001.zip › Supplementary Tables.pdf]

Supplementary Tables

Supplementary Table S1. Primer sequences and amplification characteristics for RT-qPCR

Gene	Forward primer (5'->3')	Reverse primer (5'->3')	Amplicon (bp)	Efficiency (%)	R-squared
**BDNF**	CCTTGACCCTGCAGAATGGC	GCTTTCTTTCACCGGGATGC	120 bp	98.4%	0.998
**NGF**	GGAGCGCAGCGAGTTTTG	CTTGGACTGCACGACCACT	108 bp	96.2%	0.996
**NTRK1**	GCGTCCACCTTTCTCTTCATGT	CCTTGTTCACCTGTAAGATCGTG	132 bp	92.7%	0.995
**NTRK2**	GGACCCCATCCCTGACACTA	CGGGTCTCCAGATGTGCTG	115 bp	95.8%	0.997
**ACTB**	CCTTTGCCGATCCGCCG	GATATCATCATCCATGGTGAGCTGG	146 bp	103.5%	0.999

Notes. Primers were synthesised by Thermo Fisher Scientific (Custom DNA Oligos, 25 nmol scale, desalted; supplied by ALT Ukraine Ltd., Kyiv). Specificity was verified by BLAST against the RefSeq human transcriptome and by single-peak melting-curve analysis (Tm dispersion < 0.5 degC across replicates). Amplification efficiencies were determined from 5-point 10-fold serial dilution standard curves (log10-linear regression). ACTB served as the reference gene for the 2^-DeltaDeltaCt calculation.

Supplementary Table S2. Extended baseline characteristics of the study cohort by clinical group

Characteristic	Control (n = 50)	Prediabetes (n = 50)	T2DM (n = 50)	Dep-Anx (n = 50)
**N**	50	50	50	50
**Female, n (%)**	30 (60.0%)	30 (60.0%)	26 (52.0%)	37 (74.0%)
**Male, n (%)**	20 (40.0%)	20 (40.0%)	24 (48.0%)	13 (26.0%)
**Current smokers, n (%)**	10 (20.0%)	9 (18.0%)	11 (22.0%)	10 (20.0%)
**Regular alcohol use, n (%)**	8 (16.0%)	10 (20.0%)	5 (10.0%)	3 (6.0%)
**Education, years (median, IQR)**	14.0 (12.0–15.8)	13.0 (11.0–14.0)	12.0 (11.0–13.0)	13.0 (12.0–14.0)
**Fasting insulin, uU/mL (median, IQR)**	9.3 (8.4–10.6)	19.2 (16.5–27.4)	12.8 (8.4–20.1)	9.4 (8.1–10.2)
**WHO-5 well-being score (median, IQR)**	71.5 (60.5–79.0)	56.5 (47.2–67.8)	51.0 (44.0–60.8)	39.0 (28.0–46.2)
**FINDRISC score (median, IQR)**	4.0 (2.0–6.0)	10.0 (8.0–11.0)	13.0 (11.2–14.0)	6.5 (4.0–7.8)
**PAID score (median, IQR)**	n/a	n/a	31.5 (20.0–42.0)	n/a
**-- Season of blood collection --**				
**May-October, n (%)**	23 (46.0%)	19 (38.0%)	28 (56.0%)	27 (54.0%)
**November-April, n (%)**	27 (54.0%)	31 (62.0%)	22 (44.0%)	23 (46.0%)
**-- Medications and supplements --**				
**Antidepressants (SSRI/SNRI), n (%)**	0 (0.0%)	4 (8.0%)	5 (10.0%)	35 (70.0%)
**Anxiolytics (benzodiazepines), n (%)**	0 (0.0%)	0 (0.0%)	1 (2.0%)	9 (18.0%)

**Metformin, n (%)**	0 (0.0%)	5 (10.0%)	33 (66.0%)	3 (6.0%)
**Insulin, n (%)**	0 (0.0%)	0 (0.0%)	7 (14.0%)	0 (0.0%)
**GLP-1 receptor agonists, n (%)**	0 (0.0%)	0 (0.0%)	2 (4.0%)	0 (0.0%)
**Statins, n (%)**	2 (4.0%)	14 (28.0%)	28 (56.0%)	3 (6.0%)
**Vitamin D supplementation, n (%)**	8 (16.0%)	11 (22.0%)	11 (22.0%)	12 (24.0%)

PAID (Problem Areas in Diabetes) was administered only to participants with diagnosed type 2 diabetes. WHO-5 scores are transformed to 0-100 scale (higher = better well-being). FINDRISC = Finnish Diabetes Risk Score. Season of blood collection was defined as May-October vs November-April to reflect seasonal variation in cutaneous vitamin D synthesis at the study latitude (48 degN).

Supplementary Table S2b. Clinical characteristics of the depressive-anxiety group (n = 50)

**Variable**	**Value**
**Illness duration, years (median, IQR)**	4.9 (3.2–6.2)
**ICD-10 F32 (depressive episode), n (%)**	14 (28.0%)
**ICD-10 F33 (recurrent depressive disorder), n (%)**	16 (32.0%)
**ICD-10 F41 (anxiety disorders), n (%)**	10 (20.0%)
**Comorbid depressive + anxiety diagnoses (F32+F41), n (%)**	10 (20.0%)
**Treatment-naive at inclusion, n (%)**	12 (24.0%)
**On active psychopharmacological treatment, n (%)**	38 (76.0%)

Diagnoses were established by a board-certified psychiatrist according to ICD-10 criteria. PHQ-9 and GAD-7 were used to quantify symptom severity, not as standalone diagnostic instruments. Participants with a lifetime diagnosis of bipolar disorder, schizophrenia-spectrum disorders or substance-use disorders were excluded.

Supplementary Table S3. Sensitivity analyses for the primary association between depressive symptom severity and peripheral BDNF expression

**Model**	**n**	**PHQ-9 beta (95% CI)**	**BMI beta (95% CI)**	**HbA1c beta (95% CI)**	**Adj. R-squared**
**A: Primary (manuscript model)**	200	-1.312 (-1.704, -0.921); p = < 0.001	+0.534 (+0.269, +0.799); p = < 0.001	-0.212 (-0.443, +0.018); p = 0.071	0.233
**B: + sex**	200	-1.276 (-1.651, -0.901); p = < 0.001	+0.530 (+0.266, +0.793); p = < 0.001	-0.226 (-0.459, +0.007); p = 0.057	0.235
**C: + medications**	200	-1.276 (-1.683, -0.869); p = < 0.001	+0.568 (+0.239, +0.898); p = < 0.001	-0.136 (-0.402, +0.130); p = 0.315	0.237
**D: + season**	200	-1.311 (-1.705, -0.917); p = < 0.001	+0.537 (+0.268, +0.805); p = < 0.001	-0.216 (-0.454, +0.021); p = 0.074	0.229
**E: excluding Dep-Anx (n=150)**	150	-1.212 (-1.768, -0.656); p = < 0.001	+0.407 (+0.088, +0.725); p = 0.012	-0.242 (-0.473, -0.012); p = 0.039	0.171

**F: excluding Control (n=150)**	150	-2.524 (-3.256, -1.792); p = < 0.001	-0.036 (-0.377, +0.305); p = 0.835	-0.195 (-0.420, +0.030); p = 0.090	0.293

Outcome: log(BDNF) relative expression. Predictors were z-standardised (SD = 1). All models used OLS with heteroscedasticity-consistent (HC3) standard errors. Coefficients represent the change in log(BDNF) per one standard deviation of the predictor. Model A replicates the primary multivariable model reported in the main text (Table 2). Models B-D add sex, medication use (antidepressants SSRI/SNRI, metformin, statins, vitamin D supplementation as binary covariates) and blood-collection season. Models E-F re-fit the primary model after excluding the depressive-anxiety and control groups. Across all sensitivity analyses the negative association between PHQ-9 and log(BDNF) was preserved in direction, magnitude and statistical significance.

Supplementary Table S4. Reduced peripheral BDNF expression across alternative fold-change thresholds

Threshold	Control (n = 50)	Prediabetes (n = 50)	T2DM (n = 50)	Dep-Anx (n = 50)	chi-squared	p
**1.5x (BDNF < 0.667)**	6 (12%)	7 (14%)	17 (34%)	27 (54%)	28.54	< 0.001
**2.0x (BDNF < 0.500)**	3 (6%)	7 (14%)	16 (32%)	26 (52%)	32.64	< 0.001
**3.0x (BDNF < 0.333)**	2 (4%)	7 (14%)	15 (30%)	26 (52%)	35.09	< 0.001

Values are n (%) participants whose BDNF relative expression fell below the reciprocal of the fold-change threshold. The 2x threshold (row 2) is the value reported in the main text and follows the widely used convention for biologically meaningful gene-expression change in RT-qPCR studies. Sensitivity analyses using 1.5x and 3x thresholds produced consistent between-group patterns.

Supplementary Table S5. Exploratory classification of clinically significant depression (PHQ-9 >= 15)

Metric	Full panel	Reference
**Predictors**	log(BDNF) + log(NTRK2) + 25(OH)D	25(OH)D only (reference)
**Algorithm**	L1-regularised logistic regression	L1-regularised logistic regression
**Cross-validation**	5-fold stratified	5-fold stratified
**AUC-ROC (95% CI)**	0.966 (0.946-0.983)	0.948 (0.918-0.973)
**Sensitivity**	0.924	0.924
**Specificity**	0.838	0.809
**PPV**	0.917	0.904
**NPV**	0.851	0.846
**Brier score**	0.071	0.085
**Confusion matrix (TP/TN/FP/FN)**	122/57/11/10	122/55/13/10

Outcome definition: PHQ-9 >= 15 (moderately severe to severe depression). 132 of 200 participants met this cut-off. Predictors were z-standardised prior to fitting. Cross-validated probability predictions were obtained via 5-fold stratified cross-validation; 95% confidence intervals for AUC were derived by 1000 bootstrap resamples of the out-of-fold predictions. This analysis is reported as exploratory only: because participants in the depressive-anxiety group were selected on the basis of psychiatric symptom severity, in-sample discrimination is likely optimistically biased. External validation in an independent cohort with outcomes defined independently of the group-selection procedure is required before any claim of clinical utility can be made.
