# Supplementary material for: Peripheral BDNF–TrkB Expression and Vitamin D Status in Depression, Prediabetes and Type 2 Diabetes: A Cross-Sectional Biomarker Study
Source: Biomedicines. 2026 Sep 18;14(9):2106. doi: 10.3390/biomedicines14092106 (PMC13604075; doi:10.3390/biomedicines14092106)
Supplement: Supplementary file 1 [file biomedicines-14-02106-s001.zip › Supplementary File S2.pdf]

Supplementary File S2. STROBE Statement—Checklist of items that should be included in reports of cross-sectional studies

Section / Topic	Item #	Recommendation	Reported (section / page)
Title and abstract	1(a)	Indicate the study's design with a commonly used term in the title or the abstract	Title: "…a cross-sectional biomarker study"
	1(b)	Provide in the abstract an informative and balanced summary of what was done and what was found	Abstract, structured (Background/Objectives, Methods, Results, Conclusions)
Introduction			
Background/rationale	2	Explain the scientific background and rationale for the investigation being reported	Section 1 (Introduction), paragraphs 1–3
Objectives	3	State specific objectives, including any prespecified hypotheses	Section 1 (Introduction), final paragraph; four specific research questions (i)–(iv)
Methods			
Study design	4	Present key elements of study design early in the paper	Section 2.1: "comparative cross-sectional study… 200 adult participants… four groups of 50"
Setting	5	Describe the setting, locations, and relevant dates, including periods of recruitment, exposure, follow-up, and data collection	Section 2.1: "outpatient endocrinology department of the Chernivtsi Regional Clinical Hospital from September 2024 to December 2025"
Participants	6(a)	Give the eligibility criteria, and the sources and methods of selection of participants	Section 2.1 (Inclusion and Exclusion Criteria); Figure 1 (participant flow: 312 assessed → 112 excluded → 200 included)
Variables	7	Clearly define all outcomes, exposures, predictors, potential confounders, and effect modifiers. Give diagnostic criteria, if applicable	Section 2.2 (clinical/biochemical/psychometric); Section 2.3 (RT-qPCR); Section 2.1 (medications recorded); Section 2.4.1 (primary/secondary outcomes)
Data sources/measurement	8*	For each variable of interest, give sources of data and details of methods of assessment (measurement). Describe comparability of assessment methods if there is more than one group	Section 2.2 (Cobas c311/e411 analysers, standardised assays, validated psychometric instruments); Section 2.3 (RT-qPCR platform, kits, Supplementary Table S1 for primer details)
Bias	9	Describe any efforts to address potential sources of bias	Section 2.3 (laboratory blinding, stratified randomisation across PCR plates); Section 2.4.1 (a priori sample

			size, FDR correction, pre-specified analyses); Sensitivity analyses (Discussion, Supplementary Table S3)
Study size	10	Explain how the study size was arrived at	Section 2.4.1: "n = 168 provides 90% power to detect a partial correlation of ρ = 0.25 at α = 0.05; achieved sample n = 200"
Quantitative variables	11	Explain how quantitative variables were handled in the analyses. If applicable, describe which groupings were chosen and why	Section 2.4 (z-standardisation of continuous predictors; log-transformation of BDNF; severity strata for PHQ-9/GAD-7 per instrument developers' cut-offs)
Statistical methods	12(a)	Describe all statistical methods, including those used to control for confounding	Sections 2.4 and 2.4.1 (Kruskal–Wallis + Dunn; Spearman ρ + partial correlations; OLS with HC3; Huber M-estimation; median regression; hierarchical + dominance; TOST; bootstrap; VIF)
	12(b)	Describe any methods used to examine subgroups and interactions	Section 3.8 (PHQ-9 × BMI interaction, simple slopes); Discussion + Supplementary Table S3 (within-group correlations; sensitivity analyses excluding groups)
	12(c)	Explain how missing data were addressed	Section 2.4.1 / Figure 1: "Missing data were < 2% for all analysed variables; listwise deletion"
	12(d)	If applicable, describe analytical methods taking account of sampling strategy	N/A (consecutive within-group recruitment to fixed n = 50; no complex sampling)
	12(e)	Describe any sensitivity analyses	Discussion (paragraph on sensitivity analyses) and Supplementary Table S3 (models with medications, sex, season, subgroup exclusions); Supplementary Table S4 (alternative fold-change thresholds)
Results			
Participants	13(a)	Report numbers of individuals at each stage of study (numbers potentially eligible, examined for eligibility, confirmed eligible, included in the study, completing follow-up, and analysed)	Figure 1 (CONSORT-style flow: 312 assessed → 112 excluded → 200 included → 200 analysed)
	13(b)	Give reasons for non-participation at each stage	Figure 1 caption (acute infection n = 27; oncological/autoimmune n = 22; hepatic/renal insufficiency n = 14; recent cardiovascular event n = 9; pregnancy n = 6; declined n = 34)

	13(c)	Consider use of a flow diagram	Figure 1
Descriptive data	14(a)	Give characteristics of study participants (e.g., demographic, clinical, social) and information on exposures and potential confounders	Table 1 (main cohort characteristics); Supplementary Table S2 (extended demographics, lifestyle, medications, season); Supplementary Table S2b (Dep-Anx clinical detail)
	14(b)	Indicate number of participants with missing data for each variable of interest	Section 2.4.1: "Missing data were < 2% for all analysed variables"
Outcome data	15*	Report numbers of outcome events or summary measures	Section 3.1 and Table 1 (median, IQR by group); Section 3.2 (proportions with reduced BDNF; χ^2); Figure 3
Main results	16(a)	Give unadjusted estimates and, if applicable, confounder-adjusted estimates and their precision (e.g., 95% confidence interval). Make clear which confounders were adjusted for and why they were included	Section 3.3 (unadjusted Spearman + partial correlations); Section 3.6 and Table 2 (multivariable OLS with HC3; robust and median regressions with 95% CI); Section 3.8 (interaction and simple slopes)
	16(b)	Report category boundaries when continuous variables were categorised	Section 2.2 (vitamin D status: adequate 30–50, insufficiency 20–29, deficiency < 20 ng/mL); Section 2.4 (PHQ-9/GAD-7 severity strata per instrument developers' cut-offs)
	16(c)	If relevant, consider translating estimates of relative risk into absolute risk for a meaningful time period	N/A (cross-sectional design; no time-based risk estimates)
Other analyses	17	Report other analyses done—e.g., analyses of subgroups and interactions, and sensitivity analyses	Section 3.7 (bootstrap decomposition; network analysis); Section 3.8 (dominance analysis; PHQ-9 × BMI interaction); Discussion + Supplementary Tables S3–S5 (sensitivity analyses; alternative thresholds; exploratory classifier)
Discussion			
Key results	18	Summarise key results with reference to study objectives	Discussion, opening paragraphs (BDNF/NTRK2 decline in affective phenotype; vitamin D as strongest correlate; psychiatric > metabolic contribution to BDNF variance)
Limitations	19	Discuss limitations of the study, taking into account sources of potential bias or imprecision. Discuss both direction and magnitude of any potential bias	Discussion, penultimate paragraph (cross-sectional design; whole-blood transcriptomics; single-centre; residual confounding by diet/activity/inflammation; PHQ-9/GAD-7 not standalone diagnostic instruments)

Interpretation	20	Give a cautious overall interpretation of results considering objectives, limitations, multiplicity of analyses, results from similar studies, and other relevant evidence	Discussion throughout; specifically Conclusions: "should not be interpreted as direct evidence of altered BDNF–TrkB protein signaling or causality"
Generalisability	21	Discuss the generalisability (external validity) of the study results	Discussion, closing paragraphs; classification analysis explicitly framed as "exploratory only"; external validation required (Discussion + Supplementary Table S5)
Other information			
Funding	22	Give the source of funding and the role of the funders for the present study and, if applicable, for the original study on which the present article is based	Funding statement: "This research received no external funding"

* Give information separately for exposed and unexposed groups where applicable. In the present study, information is provided for each of the four clinical groups (control, prediabetes, T2DM, depressive–anxiety) in Table 1 and Supplementary Table S2.

Note: An Explanation and Elaboration article discusses each checklist item and gives methodological background and published examples of transparent reporting. The STROBE checklist is best used in conjunction with this article (freely available on the Web sites of PLoS Medicine at http://www.plosmedicine.org/, Annals of Internal Medicine at http://www.annals.org/, and Epidemiology at http://www.epidem.com/). Information on the STROBE Initiative is available at www.strobe-statement.org.
