# Supplementary material for: Peripheral BDNF–TrkB Expression and Vitamin D Status in Depression, Prediabetes and Type 2 Diabetes: A Cross-Sectional Biomarker Study
Source: Biomedicines. 2026 Sep 18;14(9):2106. doi: 10.3390/biomedicines14092106 (PMC13604075; doi:10.3390/biomedicines14092106)
Supplement: Supplementary file 1 [file biomedicines-14-02106-s001.zip › Supplementary File S1.pdf]

Supplementary File S1. MIQE Checklist for RT-qPCR reporting

Item	E/D	Reported (value / manuscript location)
Experimental design		
Definition of experimental and control groups	E	Reported: Section 2.1 (four groups of 50 — apparently healthy controls, prediabetes, type 2 diabetes, depressive–anxiety); inclusion/exclusion criteria in Section 2.1.
Number within each group	E	Reported: n = 50 per group; total n = 200. Figure 1 (CONSORT flow).
Assay carried out by core lab or investigator's lab	D	Reported: Investigator's laboratory, Department of Microbiology, Virology, and Immunology, TNMU (Ternopil, Ukraine). Section 2.3.
Acknowledgement of authors' contributions	D	Reported: Author Contributions statement.
Sample		
Description	E	Reported: Whole peripheral venous blood, K3-EDTA tubes. Section 2.3.
Volume/mass of sample processed	D	Reported: 2.5 mL whole blood per participant.
Microdissection or macrodissection	E	N/A (whole blood).
Processing procedure	E	Reported: TRIzol reagent (Thermo Fisher Scientific, cat. 15596026) extraction; standard phase separation and isopropanol precipitation. Section 2.3.
If frozen — how and how quickly?	E	Reported: RNA aliquots stored at −80 °C within 2 h of collection; single freeze-thaw prior to reverse transcription.
If fixed — with what and how quickly?	E	N/A (fresh whole blood).
Sample storage conditions and duration	E	Reported: −80 °C for ≤ 12 months prior to reverse transcription.
Nucleic acid extraction		
Procedure and/or instrumentation	E	Reported: TRIzol reagent (Thermo Fisher Scientific, cat. 15596026), manual extraction per manufacturer's protocol. Section 2.3.
Name of kit and details of any modifications	E	Reported: TRIzol Reagent, no protocol modifications.
Source of additional reagents used	D	Reported: RevertAid First Strand cDNA Synthesis Kit (Thermo Fisher, cat. K1622); Maxima SYBR Green/ROX qPCR Master Mix 2× (cat. K0221). Section 2.3.
Details of DNase or RNAse treatment	E	Reported: On-column DNase I treatment during extraction (Thermo Fisher).
Contamination assessment (DNA or RNA)	E	Reported: No-reverse-transcription (NRT) controls included on every plate; all NRT Ct > 38 (i.e. below detection).
Nucleic acid quantification	E	Reported: NanoDrop spectrophotometry; RNA concentration measured for every sample.
Instrument and method	E	Reported: NanoDrop ND-1000 (Thermo Fisher).
Purity (A260/A280 and A260/A230)	D	Reported: A260/A280 = 1.9–2.1; A260/A230 ≥ 2.0 for all samples. Section 2.3.
Yield	D	Reported: RNA yield 1.2–3.8 µg per 2.5 mL whole blood.

RNA integrity: method / instrument	E	Reported: Microcapillary electrophoresis on Agilent 2100 Bioanalyzer.
RIN / RQI or Cq of 3' and 5' transcripts	E	Reported: RIN ≥ 7.5 for all samples.
Electrophoresis traces	D	Available on request.
Inhibition testing (Cq dilutions, spike or other)	E	Reported: Cq linearity across 10-fold dilutions verified for all assays ($R^2 \geq 0.995$).
Reverse transcription		
Complete reaction conditions	E	Reported: 1 µg total RNA in 20 µL reaction; 42 °C for 60 min, 70 °C for 5 min inactivation. Section 2.3.
Amount of RNA and reaction volume	E	Reported: 1 µg RNA / 20 µL reaction.
Priming oligonucleotide and concentration	E	Reported: Random hexamers (100 pmol per reaction), supplied with RevertAid kit.
Reverse transcriptase and concentration	E	Reported: RevertAid Reverse Transcriptase, 200 U per reaction.
Temperature and time	E	Reported: 42 °C for 60 min; 70 °C for 5 min.
Manufacturer of reagents and catalogue numbers	D	Reported: Thermo Fisher Scientific K1622 (RevertAid First Strand cDNA Synthesis Kit).
Cqs with and without RT	D	Reported: NRT Ct > 38 for all target assays (undetermined).
qPCR target information		
Gene symbol	E	Reported: BDNF, NGF, NTRK1, NTRK2, ACTB. Section 2.3, Supplementary Table S1.
Sequence accession number	E	Reported: RefSeq NM_170735.6 (BDNF); NM_002506.3 (NGF); NM_002529.4 (NTRK1); NM_006180.6 (NTRK2); NM_001101.5 (ACTB).
Amplicon length	E	Reported: 108–146 bp (Supplementary Table S1).
In silico specificity screen (BLAST, etc.)	E	Reported: BLAST against RefSeq human transcriptome; no off-target hits.
Pseudogenes, retropseudogenes or other homologs?	D	Reported: No high-similarity pseudogenes identified.
Sequence alignment	D	Not applicable (single-organism study).
Secondary structure analysis of amplicon	D	Verified; no strong secondary structures within amplicons at 60 °C.
Location of each primer by exon or intron	E	Reported: All primer pairs designed to span exon–exon junctions (avoids gDNA amplification). Supplementary Table S1.
What splice variants are targeted?	E	Reported: All principal protein-coding transcripts of each gene.
qPCR oligonucleotides		
Primer sequences	E	Reported: Supplementary Table S1 (all forward and reverse sequences 5'→3').
Location and identity of any modifications	E	None (unmodified, desalted).

Manufacturer of oligonucleotides	D	Reported: Custom DNA Oligos (Thermo Fisher Scientific, cat. 10629186); supplied by ALT Ukraine Ltd. (Kyiv).
Purification method	D	Reported: Desalted, 25 nmol scale.
qPCR protocol		
Complete reaction conditions	E	Reported: 20 µL final volume: 10 µL Maxima SYBR Green/ROX qPCR Master Mix 2×; 0.3 µM each primer; 2 µL cDNA; nuclease-free water to volume. Section 2.3.
Reaction volume and amount of cDNA/DNA	E	Reported: 20 µL total; 2 µL cDNA (equivalent to 100 ng input RNA).
Primer, Mg^{2+} and dNTP concentrations	E	Reported: 0.3 µM primers; Mg^{2+} and dNTP concentrations as supplied in Maxima master mix (proprietary).
Polymerase identity and concentration	E	Reported: Hot-Start Maxima Taq DNA Polymerase (Thermo Fisher).
Buffer/kit identity and manufacturer	E	Reported: Maxima SYBR Green/ROX qPCR Master Mix 2× (Thermo Fisher, cat. K0221).
Exact chemical composition of buffer	D	Proprietary formulation.
Additives (SYBR Green, EvaGreen, etc.)	E	Reported: SYBR Green I (intercalating dye) with ROX passive reference.
Manufacturer of plates/tubes and catalogue no.	D	Reported: MicroAmp Optical 96-well plates (Applied Biosystems).
Complete thermocycling parameters	E	Reported: 95 °C 10 min; 40 × (95 °C 15 s, 60 °C 60 s); melt curve 60→95 °C at 0.5 °C/step.
Reaction setup (manual/robotic)	D	Reported: Manual, single operator, single session per plate.
Manufacturer of qPCR instrument	E	Reported: Applied Biosystems 7500 Real-Time PCR System.
qPCR validation		
Evidence of optimisation (gradients, etc.)	D	Reported: Annealing temperature optimised at 60 °C; primer concentrations titrated.
Specificity (gel, sequencing, melt, digest)	E	Reported: Single-peak melting curves (Tm dispersion < 0.5 °C across replicates); confirmed by BLAST in silico.
For SYBR Green I: Cq of NTC	E	Reported: All NTC Ct > 38 (i.e. undetermined) for all assays.
Standard curves with slope and y-intercept	E	Reported: 5-point 10-fold serial dilution standard curves for each assay; slopes −3.24 to −3.53.
PCR efficiency calculated from slope	E	Reported: 92.7–103.5 % (Supplementary Table S1).
Confidence interval for PCR efficiency or SE	D	Reported: $R^2 \geq 0.995$ for all standard curves.
r^2 of standard curve	E	Reported: 0.995–0.999 (Supplementary Table S1).
Linear dynamic range	E	Reported: 5 $\log_{10}$ (10^2 – 10^6 template copies).
Cq variation at LOD	E	Reported: CV < 5 % within the working range (Ct 22–34).
Confidence intervals throughout the range	D	Available on request.

Evidence for limit of detection	E	Reported: LOD Ct ≈ 37; samples with Ct > 38 designated undetermined.
If multiplex — efficiency and LOD for each assay	E	N/A (singleplex assays).
Data analysis		
qPCR analysis program (source, version)	E	Reported: Applied Biosystems 7500 Software v2.3 (baseline and threshold auto-set; verified manually).
Method of Cq determination	E	Reported: Threshold-based (automatic) with manual review of every trace.
Outlier identification and disposition	E	Reported: Technical replicates with SD(Ct) > 0.5 re-run; no biological outliers excluded a priori.
Results for NTCs	E	Reported: All NTCs undetermined (Ct > 38).
Justification of number and choice of reference genes	E	Reported: ACTB selected a priori as the most widely used reference gene for whole-blood transcriptomics; stability confirmed empirically across all four groups (Kruskal–Wallis on raw Ct $p = 0.44$; CV of Ct < 3 %). Section 2.3. A single-reference-gene design is acknowledged as a limitation in the Discussion.
Description of normalisation method	E	Reported: $2^{(-\Delta\Delta Ct)}$ method (Livak & Schmittgen 2001) with ACTB as reference and the median of the healthy control group as calibrator. Section 2.3.
Number and concordance of biological replicates	D	Reported: n = 50 biological replicates per group (participants).
Number and stage (RT or qPCR) of technical replicates	E	Reported: 3 technical qPCR replicates per sample per assay; single RT per sample.
Repeatability (intra-assay variation)	E	Reported: Mean intra-assay CV of Ct < 3 %.
Reproducibility (inter-assay variation, CV)	D	Reported: Inter-run calibrator-normalised CV < 5 % across plates.
Power analysis	D	Reported: A priori — n = 168 provides 90 % power to detect $\rho = 0.25$ at $\alpha = 0.05$. Section 2.4.1.
Statistical methods for result significance	E	Reported: Kruskal–Wallis + Dunn post hoc (BH-FDR); multivariable OLS with HC3; robust regressions. Section 2.4 and 2.4.1.
Software (source, version)	E	Reported: Python 3.12 with SciPy 1.11, statsmodels 0.14, scikit-learn 1.4, pingouin 0.5. Complete code in Supplementary File S4.
Cq or raw data submission to RDML	D	Raw Cq data available from the corresponding author on reasonable request.

E = Essential (required by MIQE for publication); D = Desirable (recommended).

Reference: Bustin, S.A.; Benes, V.; Garson, J.A.; Hellemans, J.; Huggett, J.; Kubista, M.; Mueller, R.; Nolan, T.; Pfaffl, M.W.; Shipley, G.L.; et al. The MIQE Guidelines: Minimum Information for Publication of Quantitative Real-Time PCR Experiments. *Clin. Chem.* **2009**, *55*, 611–622. https://doi.org/10.1373/clinchem.2008.112797
